# Human beta defensin-3 mediated activation of β-catenin during human respiratory syncytial virus infection: interaction of HBD3 with LDL receptor-related protein 5

**DOI:** 10.3389/fmicb.2023.1186510

**Published:** 2023-06-22

**Authors:** Swechha M. Pokharel, Indira Mohanty, Charles Mariasoosai, Tanya A. Miura, Lisette A. Maddison, Senthil Natesan, Santanu Bose

**Affiliations:** ^1^Department of Veterinary Microbiology and Pathology, College of Veterinary Medicine, Washington State University, Pullman, WA, United States; ^2^College of Pharmacy and Pharmaceutical Sciences, Washington State University, Spokane, WA, United States; ^3^Department of Biological Sciences, University of Idaho, Moscow, ID, United States; ^4^Center for Reproductive Biology, College of Veterinary Medicine, Washington State University, Pullman, WA, United States

**Keywords:** respiratory syncytial virus, human beta defensin-3, pro-inflammatory response, LDL receptor-related protein-5, **β**-catenin

## Abstract

Respiratory Syncytial Virus (RSV) is a non-segmented negative-sense RNA virus belonging to the paramyxovirus family. RSV infects the respiratory tract to cause pneumonia and bronchiolitis in infants, elderly, and immunocompromised patients. Effective clinical therapeutic options and vaccines to combat RSV infection are still lacking. Therefore, to develop effective therapeutic interventions, it is imperative to understand virus-host interactions during RSV infection. Cytoplasmic stabilization of β-catenin protein results in activation of canonical Wingless (Wnt)/β-catenin signaling pathway that culminates in transcriptional activation of various genes regulated by T-cell factor/lymphoid enhancer factor (TCF/LEF) transcription factors. This pathway is involved in various biological and physiological functions. Our study shows RSV infection of human lung epithelial A549 cells triggering β-catenin protein stabilization and induction of β-catenin mediated transcriptional activity. Functionally, the activated β-catenin pathway promoted a pro-inflammatory response during RSV infection of lung epithelial cells. Studies with β-catenin inhibitors and A549 cells lacking optimal β-catenin activity demonstrated a significant loss of pro-inflammatory chemokine interleukin-8 (IL-8) release from RSV-infected cells. Mechanistically, our studies revealed a role of extracellular human beta defensin-3 (HBD3) in interacting with cell surface Wnt receptor LDL receptor-related protein-5 (LRP5) to activate the non-canonical Wnt independent β-catenin pathway during RSV infection. We showed gene expression and release of HBD3 from RSV-infected cells and silencing of HBD3 expression resulted in reduced stabilization of β-catenin protein during RSV infection. Furthermore, we observed the binding of extracellular HBD3 with cell surface localized LRP5 protein, and our *in silico* and protein–protein interaction studies have highlighted a direct interaction of HBD3 with LRP5. Thus, our studies have identified the β-catenin pathway as a key regulator of pro-inflammatory response during RSV infection of human lung epithelial cells. This pathway was induced during RSV infection via a non-canonical Wnt-independent mechanism involving paracrine/autocrine action of extracellular HBD3 activating cell surface Wnt receptor complex by directly interacting with the LRP5 receptor.

## Introduction

Respiratory Syncytial Virus (RSV) is a respiratory RNA virus causing pneumonia and bronchiolitis in infants, the elderly, and immunocompromised patients (Falsey et al., 2005; Nair et al., 2010; Griffiths et al., 2017). It is estimated that RSV infection is responsible for 30 million lower respiratory tract infections annually, primarily among children, which results in 3 million RSV infection-related hospitalizations and 200,000 death (Nair et al., 2010). Although prophylactic monoclonal antibodies (e.g., palivizumab; Romero, 2003) and antiviral agents such as ribavirin show variable clinical outcomes (Diseases, 1993), effective clinical therapeutic options to combat RSV infection are still lacking. Additionally, the RSV vaccine is currently unavailable (Hurwitz, 2011; Graham and Anderson, 2013), RSV-associated diseases like pneumonia and bronchiolitis manifest due to exaggerated inflammation in the airway (Imai et al., 2008; Murawski et al., 2009; Ruuskanen et al., 2011; Foronjy et al., 2016; Russell et al., 2017). Therefore, to develop effective therapeutic and prophylactic interventions, it is imperative to understand virus-host mechanisms during RSV infection.

Human lung epithelial cells play an important role in RSV infection since these cells are the major target of RSV during the early phases of respiratory tract infection (Kong et al., 2004; Kota et al., 2008; Hosakote et al., 2009; Chang et al., 2012; Shirato et al., 2012; Mgbemena et al., 2013; Hillyer et al., 2018; Corsello et al., 2022; Rajan et al., 2022).Productive infection of human lung epithelial cells by RSV results in the production of infectious progeny virion particles (Kong et al., 2004; Kota et al., 2008; Chang et al., 2012; Shirato et al., 2012; Mgbemena et al., 2013; Hillyer et al., 2018; Rajan et al., 2022). Although macrophages are the primary pro-inflammatory response generator during RSV infection, lung epithelial cells are the first innate immune responders in the airway since these cells are infected during the early phase of infection. Therefore, it is important to understand the virus-host mechanisms in human lung epithelial cells following RSV infection.

Canonical Wingless (Wnt)/β-catenin pathway is a well-established signaling cascade regulating the expression of Wnt target genes involved in various biological and physiological functions (Clevers, 2006; Zhan et al., 2017; Ren et al., 2021; Rim et al., 2022). Wnt is a soluble secreted extracellular protein that interacts with the cell surface Frizzled (Fzd) and LDL receptor-related proteins (LRP5 and LRP6) receptor complex to activate downstream events that prevent degradation of β-catenin protein in the cytoplasm. Stabilization of β-catenin protein results in its activation and subsequent translocation to the nucleus to act as a transcriptional activator of Wnt-responsive genes by associating with T-cell factor/lymphoid enhancer factor (TCF/LEF) transcription factors. Although many viruses, including respiratory viruses like influenza A virus, activate β-catenin via the Wnt pathway (More et al., 2018), to date, it is unknown whether RSV induces β-catenin activation and the role of any such activation in regulating host response during RSV infection.

Our study has demonstrated β-catenin activation by RSV in infected human lung epithelial A549 cells. Furthermore, we show β-catenin mediated transcriptional activity promoting pro-inflammatory response in RSV-infected A549 cells. Interestingly, in contrast to canonical Wnt ligand-dependent β-catenin pathway activation by extracellular Wnt ligands, we have uncovered a yet unknown non-canonical Wnt ligand-independent β-catenin pathway activation during RSV infection. We show extracellular human beta defensin-3 (HBD3) released from RSV-infected cells stabilizing β-catenin protein in infected cells. Furthermore, HBD3 interacted with Wnt receptor LRP5 to stabilize β-catenin protein for its activation of the β-catenin pathway in RSV-infected cells. Thus, in our current study, we have identified extracellular HBD3 as an “alarmin” molecule stimulating Wnt-independent β-catenin pathway in RSV-infected cells for triggering a pro-inflammatory response.

## Results

### Respiratory syncytial virus stabilizes β-catenin protein to activate β-catenin mediated transcriptional activation

Wnt/ β-catenin canonical pathway is activated following the activation of the Fzd/LRP receptor complex by extracellular Wnt ligand (Clevers, 2006; Zhan et al., 2017; Ren et al., 2021; Rim et al., 2022). This event prevents the degradation of β-catenin protein. Accumulation of β-catenin due to its stabilization results in its translocation to the nucleus. In the nucleus, β-catenin interacts with TCF/LEF transcription factors to transactivate Wnt-responsive genes. So far, it is still unknown whether RSV activates the β-catenin pathway. Therefore, we investigated whether RSV stabilizes β-catenin protein leading to activation of TCF/LEF transcription factor-driven transactivation of Wnt-target genes.

**Figure 1:**
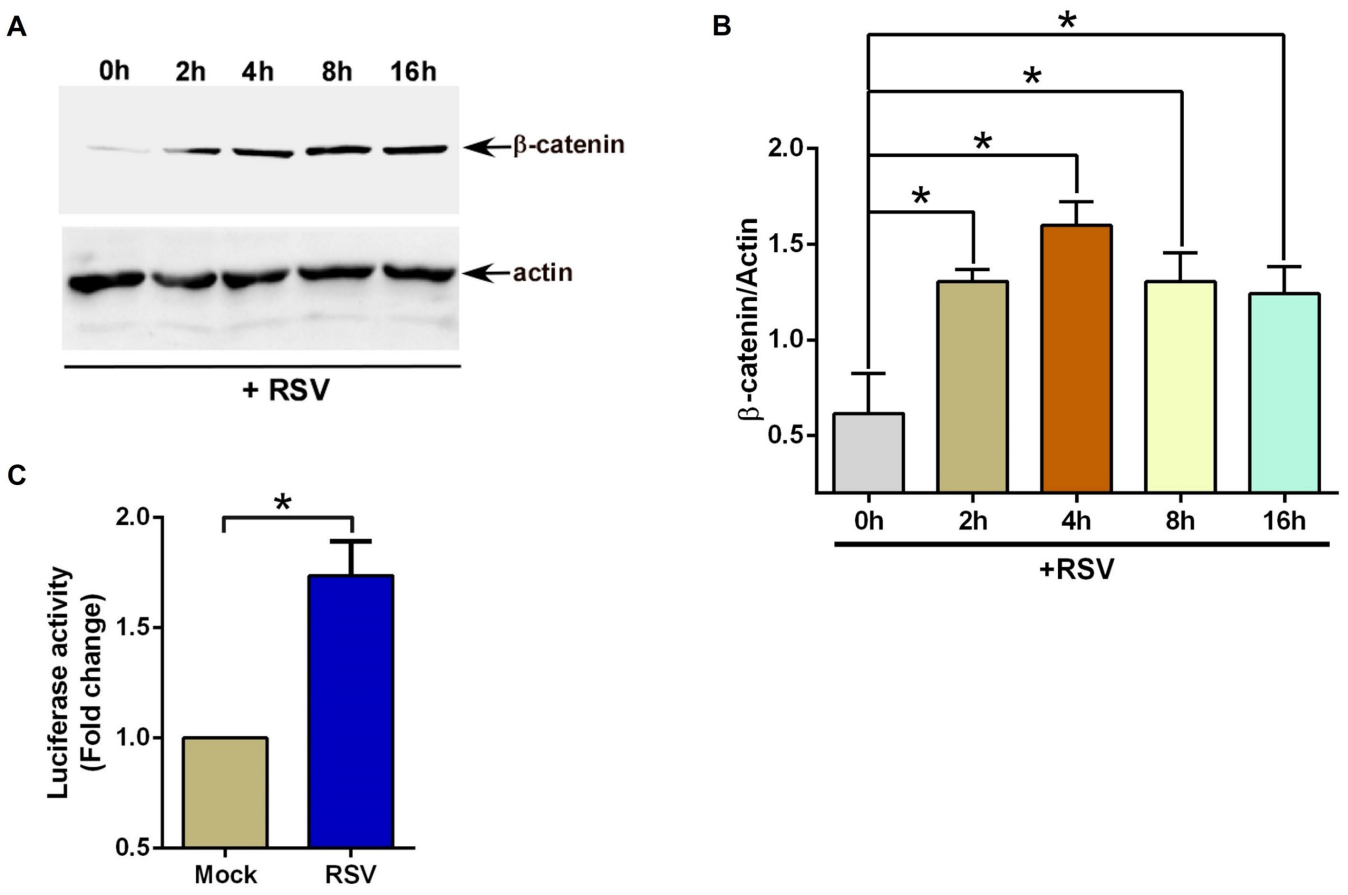
RSV induces β-catenin during infection of lung epithelial cells. **(A)** Human lung epithelial A549 cells were infected with RSV (MOI = 1) for 0–16 h. β-catenin and actin levels were determined in cell lysates by western blotting using corresponding antibodies. **(B)** Densitometry analysis of β-catenin protein levels relative to actin protein (β-catenin/Actin) in RSV-infected A549 cells. **(C)** TOP-Flash luciferase assay of A549 cells co-transfected with firefly-luciferase-TOP-Flash and renilla-luciferase plasmids. Co-transfected cells were infected with RSV (MOI = 1) for 16 h. A dual luciferase reagent was utilized to determine firefly and renilla luciferase activity. The relative TOP-Flash luciferase activity was calculated based on the mean value of firefly/renilla luciferase activity. The value is represented as a fold change in TOP-Flash activity in RSV-infected cells compared to mock infected cells. The densitometric values represent the mean ± SEM from three independent studies (*p ≤ 0.05). Luciferase assay represents mean ± SEM from three independent experiments performed in triplicates [*p ≤ 0.05 (*n* = 24; technical replicates)].

First, we investigated whether RSV infection triggers β-catenin protein stabilization since this event results in β-catenin-mediated transcriptional activation of Wnt-responsive genes. To evaluate the status of β-catenin protein, we infected human lung epithelial A549 cells with RSV. At various post-infection time-periods, the level of β-catenin protein was assessed by western blotting with anti-β-catenin antibody. RSV infection triggered β-catenin protein stabilization since enhanced levels of β-catenin protein were detected in RSV infected cells (Figures 1A,B).

We next examined whether the accumulation of β-catenin protein in RSV-infected cells resulted in the activation of TCF/LEF transcription factors. For these studies, we transfected A549 cells with TOP-Flash luciferase reporter plasmid. TOP-Flash luciferase reporter contains a promoter for binding (and activation) of activated TCF/LEF transcriptional factors upstream of the luciferase gene (Lin et al., 2016; Lee et al., 2022). Therefore, TOP-Flash luciferase reporter is widely used to study transcriptional activation by β-catenin. A549 cells transfected with TOP-Flash reporter were infected with RSV, followed by luciferase assay analysis of the cell lysate. In accordance with enhanced accumulation of β-catenin protein (Figures 1A,B), RSV also activated β-catenin-dependent transcriptional activity since RSV infection resulted in luciferase gene expression via TCF/LEF transcription factor-dependent promoter activity (Figure 1C). These results suggested RSV mediated enhanced stability and accumulation of β-catenin protein and subsequent activation of β-catenin mediated transcriptional activity in infected lung epithelial cells.

### β-Catenin activity is required for an optimal pro-inflammatory response during RSV infection

Compared to macrophages, human lung epithelial cells like A549 cells do not robustly produce pro-inflammatory cytokines like TNF, IL-6, and IL-1β. Instead, RSV-infected A549 cells trigger efficient production of pro-inflammatory chemokine IL-8 (in humans, IL-8 is encoded by the *CXCL8* gene; Fiedler et al., 1995; Thomas et al., 2000; Rudd et al., 2005). IL-8 is a major neutrophil chemoattractant (Baggiolini et al., 1989; Bazzoni et al., 1991), and neutrophils are implicated in the development of exaggerated RSV-associated lung disease in children and infants (Everard et al., 1994; McNamara et al., 2003; Emboriadou et al., 2007; Sebina and Phipps, 2020). High levels of IL-8 have been detected in infants with severe RSV-associated bronchiolitis (Biswas et al., 1995). Furthermore, IL-8 serves as a biomarker for RSV-associated lung disease severity (Bont et al., 1999). Therefore, we evaluated the pro-inflammatory response in RSV-infected human lung epithelial A549 cells by analyzing the production of IL-8 following the blocking of β-catenin activity by two widely used β-catenin inhibitors, iCRT3, and iCRT14 (Gonsalves et al., 2011; Trujano-Camacho et al., 2021). These inhibitors block β-catenin-mediated transcriptional activity by inhibiting the interaction of β-catenin with TCF/LEF transcription factors.

**Figure 2:**
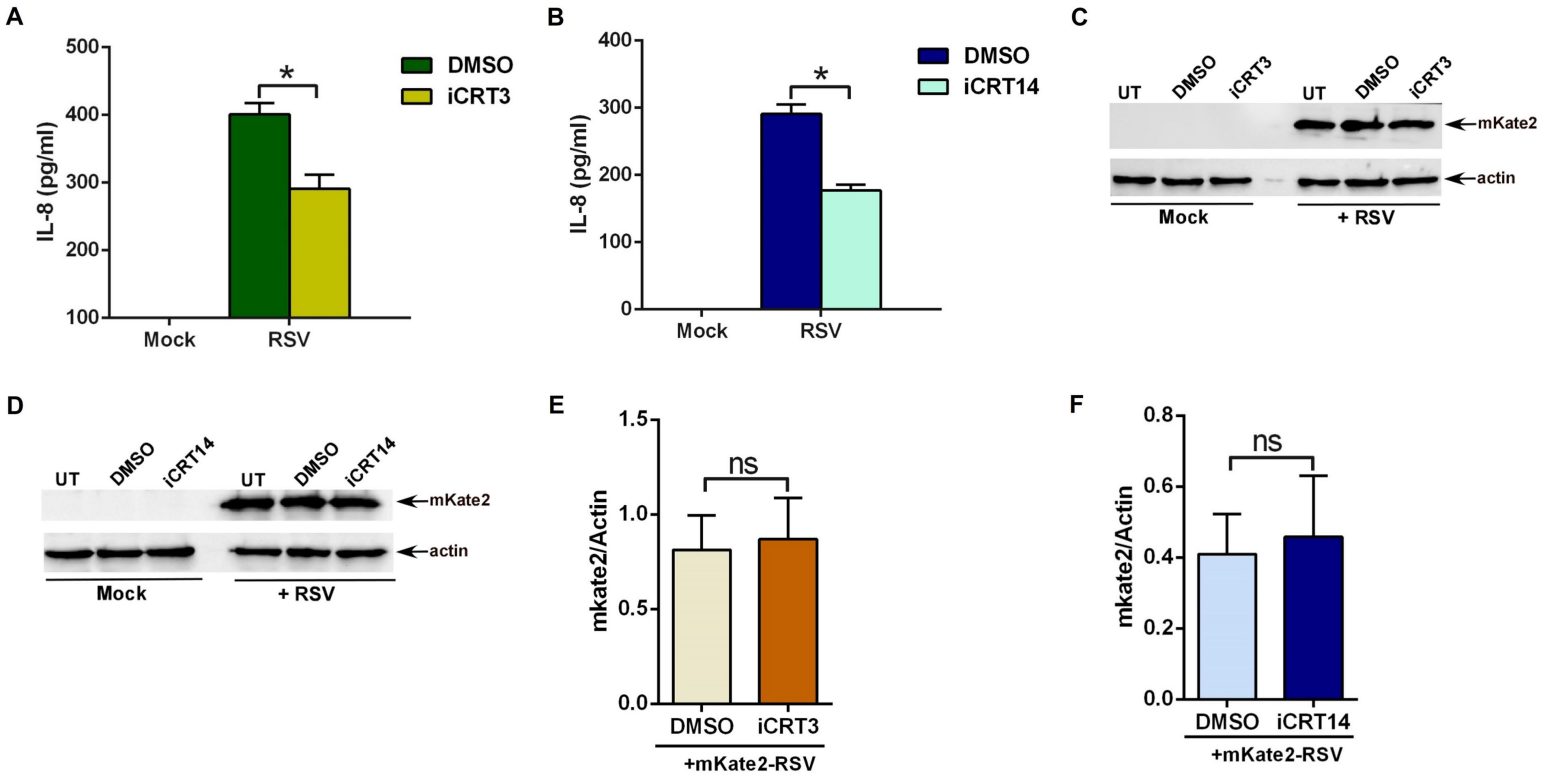
β-catenin activity is required for an optimal pro-inflammatory response during RSV infection. **(A)** Human lung epithelial A549 cells were infected with RSV (MOI = 3) in the presence of either DMSO (vehicle control) or β-catenin inhibitor iCRT3 (25 μM). Medium supernatant collected from these cells was analyzed for IL-8 production by ELISA. **(B)** A549 cells were infected with RSV (MOI = 3) in the presence of either DMSO (vehicle control) or β-catenin inhibitor iCRT14 (25 μM). Medium supernatant collected from these cells was analyzed for IL-8 production by ELISA. **(C)** A549 cells were infected with recombinant RSV expressing mKate2 protein (RSV-mKate2; MOI = 1) in the presence of either DMSO (vehicle control) or β-catenin inhibitor iCRT3 (25 μM). Cell lysate collected from these cells was subjected to western blotting with an anti-RFP antibody to detect the mKate2 protein. **(D)** A549 cells were infected with RSV-mKate2 (MOI = 1) in the presence of either DMSO (vehicle control) or β-catenin inhibitor iCRT14 (25 μM). Cell lysate collected from these cells was subjected to western blotting with an anti-RFP antibody to detect the mKate2 protein. **(E,F)** Densitometry analysis of mKate2 protein levels relative to actin protein (mKate2/Actin) in RSV-mKate2 infected A549 cells treated with either DMSO or β-catenin inhibitors (iCRT3 and iCRT14). ELISA data are shown as Mean ± SEM [*p ≤ 0.05 (*n* = 22; technical replicates; three independent experiments)]. The densitometric values represent the mean ± SEM from three independent studies (*p ≤ 0.05). ns; non-significant.

To assess the role of β-catenin activity, A549 cells were treated with either DMSO (vehicle control) or β-catenin inhibitors (iCRT3 and iCRT14) during RSV infection. Medium supernatant was collected from infected cells to analyze IL-8 production by ELISA. β-catenin activity is essential for an optimal pro-inflammatory response during RSV infection, since significant loss of IL-8 production was observed in cells treated with β-catenin inhibitors (Figures 2A,B). Approximately 50% loss of pro-inflammatory response in RSV-infected cells was noted following β-catenin activity inhibition (Figures 2A,B). To confirm that diminished pro-inflammatory response is not due to reduced RSV infection, we used recombinant RSV expressing mKate2 protein (mKate2-RSV; Bedient et al., 2020). Treatment of cells with β-catenin inhibitors did not alter RSV replication/infection as deduced by western blot analysis of cell lysate with anti-RFP antibody, which detects mKate2 protein (Figures 2C–F). These results demonstrated an important role of β-catenin activity in supporting optimal pro-inflammatory response during RSV infection of human lung epithelial A549 cells.

### β-Catenin expression is required for an optimal pro-inflammatory response during RSV infection

Since blocking β-catenin activity resulted in dampened pro-inflammatory response during RSV infection (Figures 2A,B), we further validated these results by using CRISPR-Cas9 genome-editing (Ran et al., 2013; Hsu et al., 2014; Musunuru, 2017; Hillary and Ceasar, 2022; Wang et al., 2022; Zhu, 2022) to obtain stable A549 cells lacking optimal β-catenin activity. We decided to generate a truncated version of β-catenin since previous studies have highlighted the existence of a compensatory mechanism in case of complete loss of β-catenin protein in cells (Liu et al., 2022). Several homologs exist for the Wnt/β-catenin pathway, which gives rise to redundancy to fall back on an alternative cellular pathway (Liu et al., 2022). To avoid this complication, we obtained a cell line with truncated β-catenin protein rather than generating β-catenin null cells.

**Figure 3:**
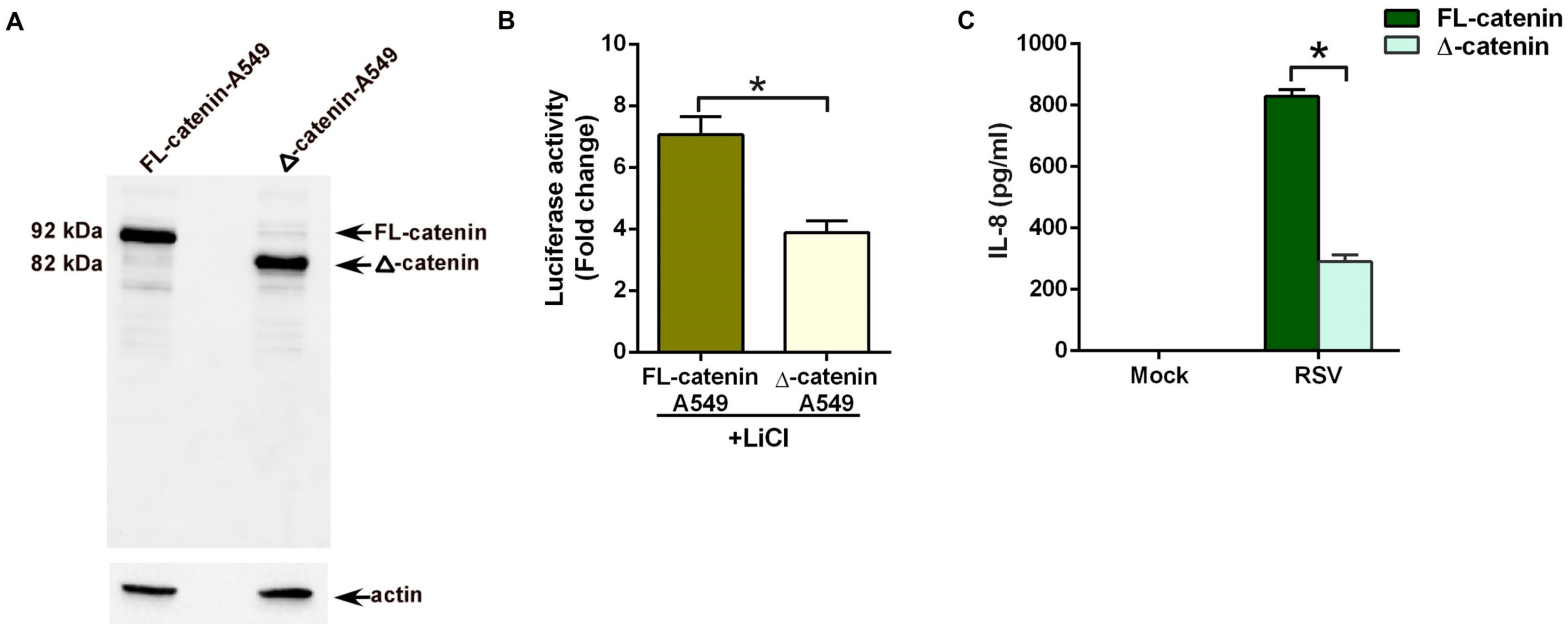
Reduced pro-inflammatory response during RSV infection of lung epithelial cells lacking full-length β-catenin protein. **(A)** CRISPR-Cas9 technology was used to generate stable human lung epithelial A549 cells lacking full-length β-catenin protein. Cell lysates from A549 cells expressing full-length 92 kDa β-catenin protein (FL-catenin-A549 cells) and A549 cells expressing truncated 82 kDa β-catenin protein (Δ-catenin-A549 cells) were subjected to western blotting with β-catenin antibody. **(B)** TOP-Flash luciferase assay of A549 cells expressing either FL-catenin or Δ-catenin were co-transfected with firefly-luciferase-TOP-Flash and renilla-luciferase plasmids. Co-transfected cells were treated with Lithium Chloride (LiCl; 25 mM) for 24 h. A dual luciferase reagent was utilized to determine firefly and renilla luciferase activity. The relative TOP-Flash luciferase activity was calculated based on the mean value of firefly/renilla luciferase activity. The value is represented as a fold change in TOP-Flash activity in LiCl-treated cells compared to vehicle (water) treated cells. **(C)** A549 cells expressing either FL-catenin or Δ-catenin were infected with RSV (MOI = 3). Medium supernatant collected from these cells was analyzed for IL-8 production by ELISA. Luciferase assay represents mean ± SEM from two independent experiments performed in triplicates [*p ≤ 0.05 (*n* = 16; technical replicates)]. ELISA data are shown as Mean ± SEM [*p ≤ 0.05 (*n* = 24; technical replicates; three independent experiments)].

The typical strategy for employing CRISPR/Cas9 to disrupt a gene has been to target near the 5′ end and isolate a mutation that should lead to a frameshift and premature termination codon and no protein would be expected (Hsu et al., 2014). Instead of such a null cell line, we isolated a clone harboring a 425 bp deletion which removes part of intron 2 extending into exon 3 (Supplementary Figure S1). Western blot analysis showed full-length (FL) 92 kDa β-catenin protein in wild-type (FL-catenin A549cells) control cells expressing control or scrambled guide-RNA (gRNA; Figure 3A). In contrast, a truncated 82 kDa β-catenin protein was detected in A549 cells with the CRISPR induced deletion (Δ-catenin A549 cells; Figure 3A). The truncated β-catenin protein observed on the western blot is the likely result of the 425 bp deletion, with splicing expected from exon 2 to exon 4 (Supplementary Figure S1). This would result in a CTNNB1 (CTNNB1 gene encodes β-catenin protein) isoform of approximately 10 kDa less than the wild-type and lacking the 77 amino acids encoded by exon 3.

To validate the loss of β-catenin activity in Δ-catenin cells, we transfected FL-catenin and Δ-catenin cells with TOP-Flash luciferase reporter plasmid. Transfected cells were then treated with lithium chloride (LiCl), a potent inducer of β-catenin-mediated transcriptional activity (Clément-Lacroix et al., 2005; Lin et al., 2016; Lee et al., 2022). Luciferase assay revealed robust induction of β-catenin mediated transcriptional activity in FL-catenin cells (Figure 3B). In contrast, significant loss of such activity (reduction by 50%) was noted in LiCl treated Δ-catenin cells (Figure 3B). This result demonstrated dampened β-catenin dependent transcriptional activity in Δ-catenin cells.

Next, we investigated whether β-catenin activity is required for a pro-inflammatory response during RSV infection. For these studies, we infected FL-catenin and Δ-catenin cells with RSV. As expected, RSV triggered a pro-inflammatory response in FL-catenin cells since high levels of IL-8 was produced from infected FL-catenin cells (Figure 3C). In contrast, drastic loss (reduction by 75%) of IL-8 production was observed in RSV infected Δ-catenin cells (Figure 3C). These results demonstrated a key requirement of β-catenin activity for a pro-inflammatory response during RSV infection of lung epithelial cells.

### Human beta-defensin 3 stabilizes β-catenin protein to activate the β-catenin-mediated transcriptional activation

To identify non-canonical extracellular ligands for activation of the β-catenin pathway via a Wnt-independent mechanism, we focused our attention on human beta-defensins or HBDs. HBDs (primarily HBD1-3) are cationic anti-microbial peptides derived from mucosal epithelial cells (Fruitwala et al., 2019; Xu and Lu, 2020). HBDs are released to the extracellular milieu upon infection, and they constitute an important innate defense factor against invading pathogens, including viruses. We have previously shown that single-stranded non-segmented RNA viruses like RSV (Kota et al., 2008) and VSV (vesicular stomatitis virus; Basu et al., 2010) triggers the release of HBD2 and HBD3, respectively, in the extracellular milieu and these HBDs block viral cellular entry. More importantly, one study reported β-catenin activation by HBD3 in human non-epithelial dental cells (human periodontal ligament cells; Zhou et al., 2018). Based on these previous studies, we next investigated whether extracellular HBD3 is capable of modulating β-catenin protein stability and β-catenin-associated transcriptional activity in lung epithelial cells.

**Figure 4:**
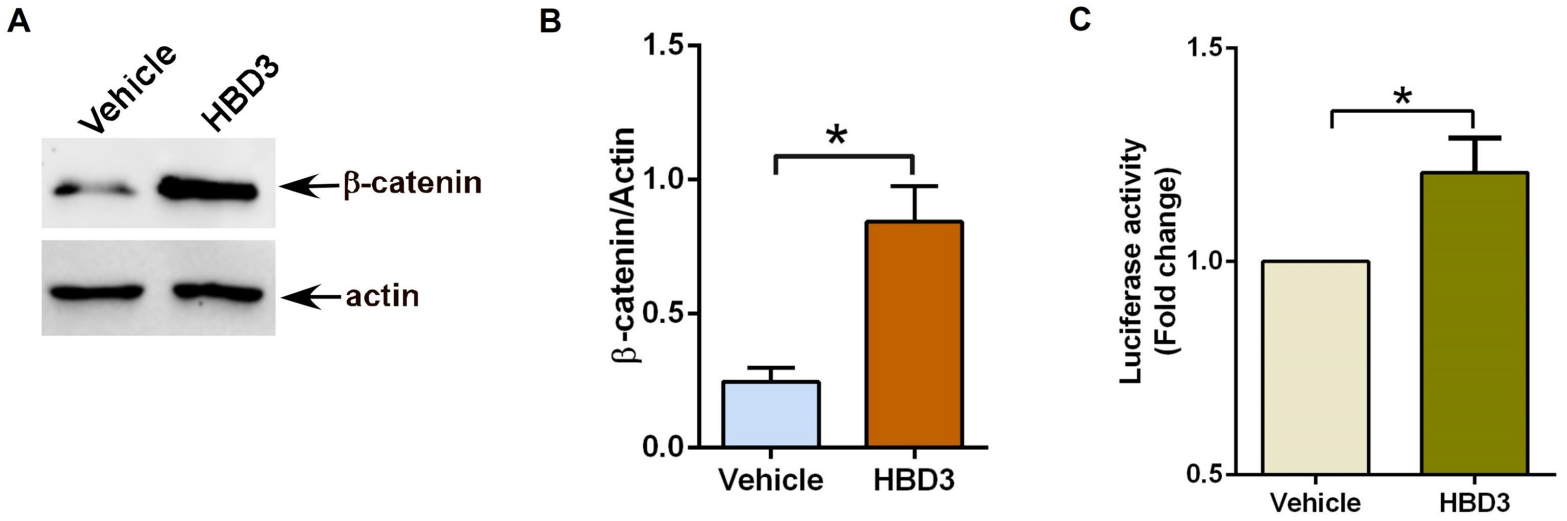
Human defensin-3 induces β-catenin activity in lung epithelial cells. **(A)** Human lung epithelial A549 cells were treated with either vehicle (0.1% BSA in PBS) or purified human defensin-3 (HBD3) protein (10 μg/ml) for 16 h. β-catenin and actin levels were determined in cell lysates by western blotting using corresponding antibodies. **(B)** Densitometry analysis of β-catenin protein levels relative to actin protein (β-catenin/Actin) in the vehicle and HBD3-treated A549 cells. **(C)** TOP-Flash luciferase assay of A549 cells co-transfected with firefly-luciferase-TOP-Flash and renilla-luciferase plasmids. Co-transfected cells were treated with either vehicle (0.1% BSA in PBS) or HBD3 for 16 h. A dual luciferase reagent was utilized to determine firefly and renilla luciferase activity. The relative TOP-Flash luciferase activity was calculated based on the mean value of firefly/renilla luciferase activity. The value is represented as a fold change in TOP-Flash activity in HBD3-treated cells compared to vehicle-treated cells. The densitometric values represent the mean ± SEM from three independent studies (*p ≤ 0.05). Luciferase assay represents mean ± SEM from two independent experiments performed in triplicates [*p ≤ 0.05 (*n* = 6; technical replicates)].

We first evaluated the ability of purified HBD3 to activate the β-catenin pathway by stabilizing cytoplasmic β-catenin protein. For these studies, we treated A549 cells with purified HBD3 (10 μg/ml) for 16 h. Previous studies showed that treating A549 cells with more than 20 μg/ml of purified HBD3 protein triggers cytotoxicity (Su et al., 2018). Therefore, we used 10 μg/ml of HBD3 protein to ensure cell viability. Treated cells were subjected to western blot analysis with anti-β-catenin antibody. HBD3 treatment led to enhanced β-catenin protein levels due to its stabilization (Figures 4A,B). Next, we investigated whether purified HBD3 triggers β-catenin-dependent transcriptional activity in A549 cells. A549 cells transfected with TOP-Flash luciferase reporter plasmid were treated with purified HBD3 for 16 h. Subsequent luciferase assay with cell lysates revealed activation of β-catenin-mediated transcriptional activity by HBD3 (Figure 4C). Thus, we have identified HBD3 as an extracellular ligand involved in Wnt ligand-independent β-catenin activation in lung epithelial cells.

### HBD3 is induced/released during RSV infection of human lung epithelial cells

**Figure 5:**
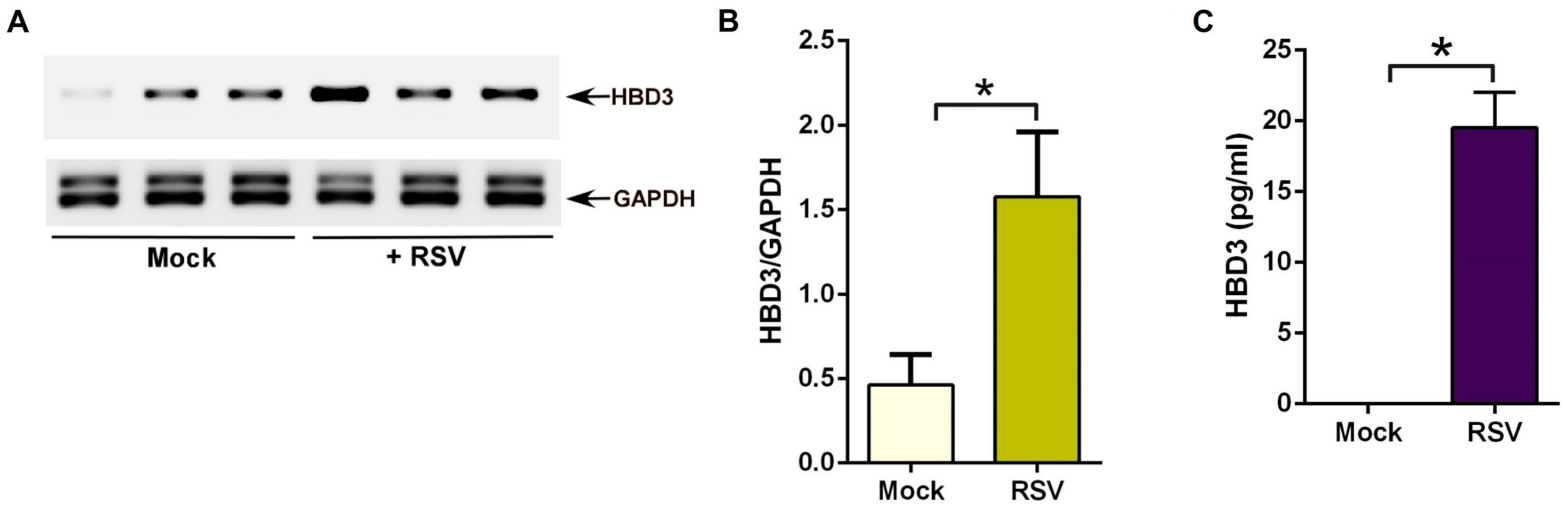
RSV induces HBD3 expression and release from lung epithelial cells. **(A)** Human lung epithelial A549 cells were infected with RSV (MOI = 1) for 16 h. RNA isolated from these cells was subjected to RT-PCR to analyze the expression of HBD3 and GAPDH (loading control). The PCR from three independent experiments (each lane corresponds to each independent experiment) are shown in the mock-infected and RSV-infected panels. **(B)** Densitometry analysis of HBD3 mRNA levels relative to GAPDH mRNA (HBD3/GAPDH) in mock vs. RSV-infected (16 post-infection) A549 cells. **(C)** Medium supernatant collected from RSV-infected (MOI = 1; 16 h post-infection) A549 cells were analyzed for HBD3 release by ELISA. The densitometric values represent the mean ± SEM from three independent studies (*p ≤ 0.05). ELISA data are shown as Mean ± SEM [*p ≤ 0.05 (*n* = 18; technical replicates; three independent experiments)].

Similar to RSV-infected cells (Figure 1), HBD3 also activated β-catenin-mediated transcriptional activity (Figure 4). Therefore, we next investigated the possibility of RSV inducing HBD3 expression and its release from infected cells to stabilize β-catenin protein via autocrine/paracrine action. To examine HBD3 expression during RSV infection, we assessed HBD3 mRNA levels in A549 cells during RSV infection. Although HBD3 is expressed at basal levels in uninfected cells, RSV infection induced HBD3 expression, since PCR analysis revealed enhanced levels of HBD3 transcripts in RSV-infected A549 cells compared to mock-infected cells (Figures 5A,B). The enhanced expression also resulted in HBD3 release into the extracellular milieu following RSV infection as determined by performing ELISA analysis with medium supernatant obtained from RSV-infected A549 cells (Figure 5C). These results demonstrated RSV-mediated induction of HBD3 expression for its release from infected lung epithelial cells.

### HBD3 is involved in β-catenin protein stabilization during RSV infection

**Figure 6:**
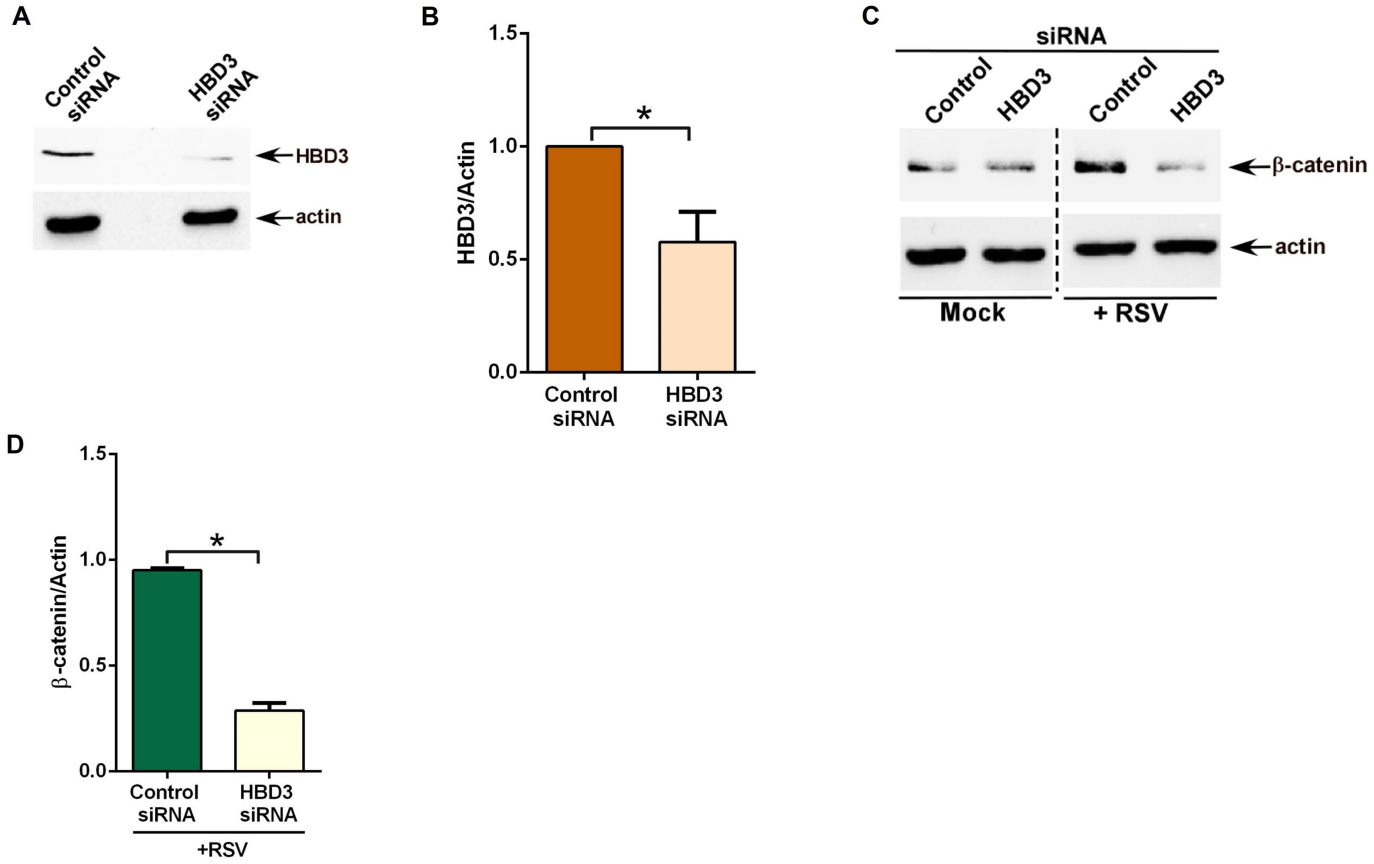
HBD3 promotes β-catenin protein stabilization during RSV infection of lung epithelial cells. **(A)** Human lung epithelial A549 cells were transfected with either control scrambled siRNA or siRNA specific for HBD3. HBD3 and actin levels were determined in cell lysates of siRNA-transfected cells by western blotting using corresponding antibodies. **(B)** Densitometry analysis of HBD3 protein levels relative to actin protein (HBD3/Actin) in control siRNA and HBD3 siRNA transfected A549 cells. **(C)** Control siRNA and HBD3 siRNA transfected cells were infected with RSV (MOI = 1) for 16 h. β-catenin and actin levels were determined in cell lysates by western blotting using corresponding antibodies. **(D)** Densitometry analysis of β-catenin protein levels relative to actin protein (β-catenin/Actin) in RSV-infected A549 cells transfected with either control siRNA or HBD3 siRNA. The densitometric values represent the mean ± SEM from three independent studies (*p ≤ 0.05).

Next, we investigated whether HBD3 plays any role in modulating β-catenin protein stability during RSV infection. We focused on β-catenin protein stability since stabilization of β-catenin protein results in activation of β-catenin mediated transcriptional activity, including expression/production of pro-inflammatory mediator IL-8. For these studies, we silenced HBD3 expression in A549 cells by siRNA. Efficient silencing was evident from western blot analysis showing reduced HBD3 protein levels in cells transfected with HBD3-specific siRNA compared to cells transfected with control scrambled siRNA (Figures 6A,B). HBD3 silenced cells were subsequently infected with RSV to evaluate β-catenin protein status during infection. As expected, RSV infection resulted in enhanced β-catenin protein levels due to its stabilization during infection of control siRNA transfected cells (Figure 6C). Interestingly, such stabilization of β-catenin protein was lacking in RSV infected cells silenced for HBD3 expression (Figures 6C,D). These results demonstrated the role of HBD3 in stabilizing β-catenin protein during RSV infection. Thus, HBD3 acts as a non-Wnt ligand to stabilize β-catenin protein for triggering β-catenin-mediated transcriptional activity in RSV-infected lung epithelial cells.

### Interaction of HBD3 with LRP5

We have identified HBD3 as a ligand involved in β-catenin activation during RSV infection. Therefore, we next investigated the mechanism by which HBD3 may confer its β-catenin activation function. Nineteen different Wnt ligands engage with cell surface receptor complexes comprising of 10 different frizzled receptors (Fzd) and LRP5/6 to activate β-catenin by triggering its stabilization (Clevers, 2006; Zhan et al., 2017; Ren et al., 2021; Rim et al., 2022). In the absence of Wnt signaling, β-catenin associates with the dissociation complex (Axin, GSK-3, APC, CK1) to undergo ubiquitin-proteasome mediated degradation. However, binding of Wnt ligands to the Fzd/LRP receptor complex transduces a signal to disrupt the dissociation complex, and as a result, β-catenin protein is stabilized due to loss of its degradation via the proteasome. Subsequently, β-catenin is targeted to the nucleus for transactivation of Wnt-responsive genes.

**Figure 7:**
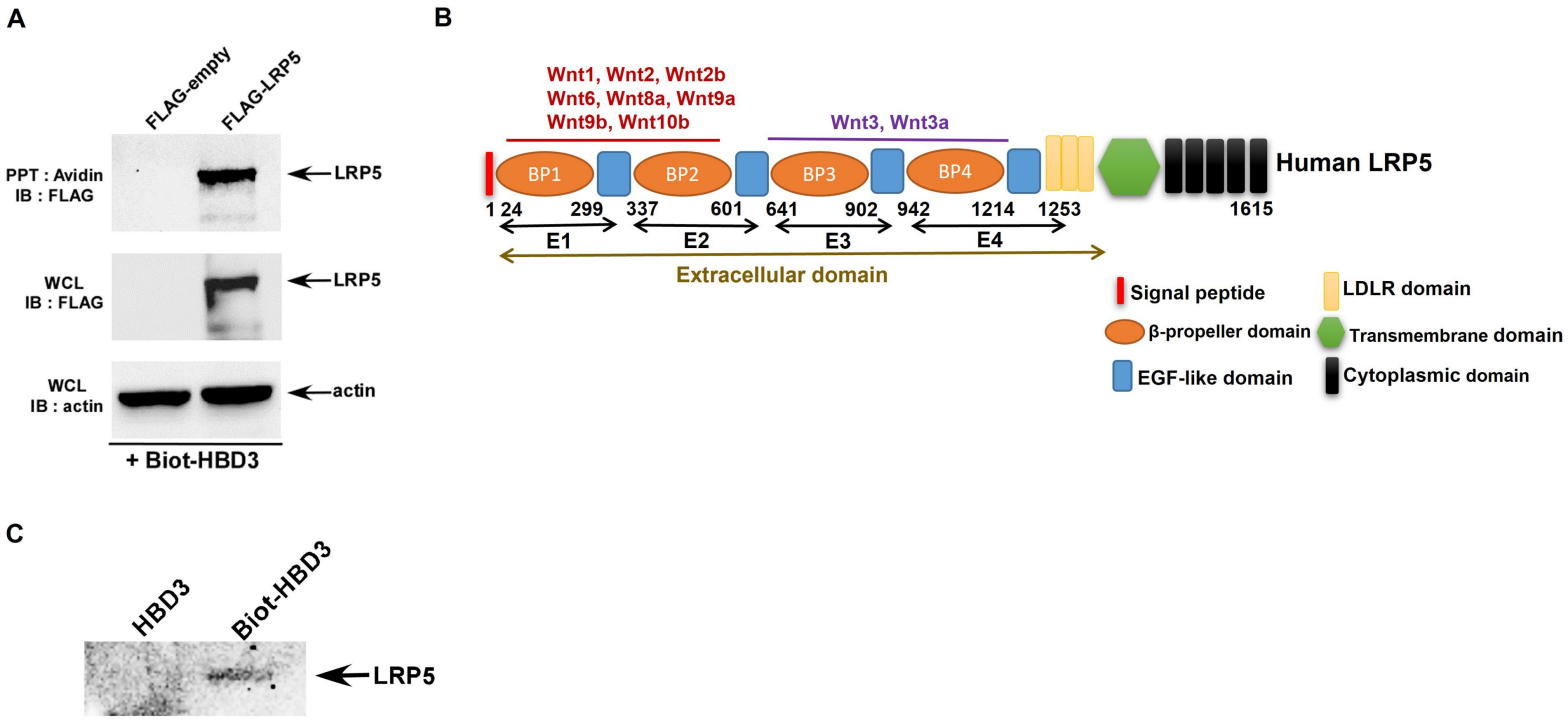
Interaction of HBD3 with LRP5. **(A)** Human lung epithelial A549 cells were transfected with either empty FLAG plasmid (FLAG-empty) or plasmid encoding FLAG-tagged LRP5 protein (FLAG-LRP5). Chilled FLAG-empty and FLAG-LRP5 A549 cells were incubated with biotinylated purified HBD3 protein (Biotin-HBD3) at 4°C for 4 h. Following incubation, cell lysates were precipitated (PPT) with avidin-agarose and subsequently immuno-blotted (IB) with FLAG antibody to detect LRP5. Whole-cell lysates (WCL) were also blotted with FLAG and actin antibodies. **(B)** A schematic showing the domain structure of the LRP5 protein. **(C)** Biot-HBD3 and non-biotinylated HBD3 (control) were precipitated (PPT) with avidin-agarose. The avidin-agarose was then incubated with purified truncated LRP5 protein comprising of the E3 and E4 domains of the extracellular domain of LRP5. Following incubation, the agarose-avidin-bound protein was subjected to immunoblotting with the LRP5 antibody. The immune blots are representative of three independent experiments with similar results.

To identify whether HBD3 can interact with Wnt receptors, we first evaluated the plausible interaction of HBD3 with LRP5. We chose LRP5 since, unlike human Fzd receptors, which consist of 10 structurally related proteins, Wnt can trigger signaling by interacting with LRP5 in complex with one of the Fzd receptors. We initially examined the possible interaction of extracellular HBD3 with cell surface localized LRP5 by using purified biotinylated-HBD3 protein (biot-HBD3). The interaction of extracellular HBD3 with cell surface localized LRP5 was studied by incubating chilled (to prevent internalization of biot-HBD3 added extracellularly) A549 cells over-expressing FLAG-tagged LRP5 protein with biot-HBD3. After incubation, cell lysate precipitated with avidin-agarose, was subjected to western blotting with anti-FLAG antibody to detect FLAG-LRP5. HBD3 interacted with cell surface LRP5, since immune-blotting of avidin-precipitated lysate with anti-FLAG antibody detected FLAG-LRP5 protein (Figure 7A). In contrast, such interaction was not noted in control cells expressing empty FLAG plasmid (Figure 7A).

Since we observed the interaction of HBD3 with cell surface LRP5, we next evaluated whether this interaction constitutes direct binding between two proteins. This study was important since well-established Wnt ligands directly interact with LRP5 protein. LRP5 protein is a large 180 kDa protein comprising several distinct extracellular domains, including four β-propeller domains (P1, P2, P3, P4), four EGF-like domains (E1, E2, E3, E4), and three LDLR type A domains (Figure 7B). Four β-propeller domains (P1, P2, P3, P4) and four EGF-like domains (E1, E2, E3, E4) are tandemly located next to each other and are designated as PE1, PE2, PE3, and PE4 domains (Figure 7B). Wnt1, Wnt2, Wnt2b, Wnt6, Wnt8a, Wnt9a, Wnt9b, Wnt10b interacts with PE1and PE2 domain of LRP5, whereas Wnt3, Wnt3a binds to the P3 domain of LRP5 to activate β-catenin signaling. We first examined whether HBD3 interacts with the PE3 extracellular domain of LRP5. For these studies, we used truncated recombinant purified LRP5 protein (aa769–aa1016, 28 kDa) encompassing majority of the PE3 (major portion of P3 and full portion of E3) domain and partial segment of the PE4 (only the N-terminal portion of P4) domain of LRP5 protein (Figure 7B). For cell-free *in vitro* interaction assay, we incubated purified non-biotinylated HBD3 (control) and biotinylated-HBD3 proteins with avidin-agarose. Subsequently, avidin-agarose beads were incubated with recombinant purified truncated LRP5 protein. Following incubation, the bound proteins were subjected to western blotting with an anti-LRP5 antibody. Our studies revealed direct interaction of HBD3 with LRP5 since we detected the HBD3-LRP5 complex bound to the agarose beads (Figure 7C). Interestingly, HBD3 interacted with the same region of LRP5 protein involved in binding to the Wnt and Wnt3a ligand. Thus, our studies have identified HBD3 as a new ligand for the LRP5 receptor involved in β-catenin activation. We also show that the PE3 domain of LRP5 is involved in interaction with HBD3.

### *In silico* studies of HBD3-LRP5 interaction

Since HBD3 directly interacted with LRP5, we next performed a series of *in silico* modeling studies to obtain detailed information about the LRP5-HBD3 interaction.

#### Modeling the binary (LRP5-HBD3) and ternary (LRP5-HBD3-FZD) complexes

Wnt ligands interact promiscuously with 10 FZD receptors and two LRPs (LRP5 and LRP6), and each Wnt ligand has the potential to activate several pairs of LRP and FZD receptors (Janda et al., 2012; Dijksterhuis et al., 2015). Previous studies have revealed that the Wnt ligands are likely sandwiched between the LRP5 and FZD8 receptors to form the LRP5-Wnt-FZD8 heterotrimer interaction complex (Hirai et al., 2019). As our study reveals that HBD3 activates the β -catenin pathway through interactions with LRP5, we hypothesize that HBD3 acts like a Wnt ligand in a manner similar to other known Wnt ligands. Therefore, we used the LRP5-Wnt-FZD8 ternary interaction model as a template to generate the plausible binary (LRP5-HBD3) and ternary (LRP5-HBD3-FZD8) complexes. To generate such a model, initially, HBD3 was docked to LRP5, and the resulting LRP5-HBD3 complex was subsequently docked to the FZD8 receptor.

#### Docking of LRP5 and HBD3

**Figure 8:**
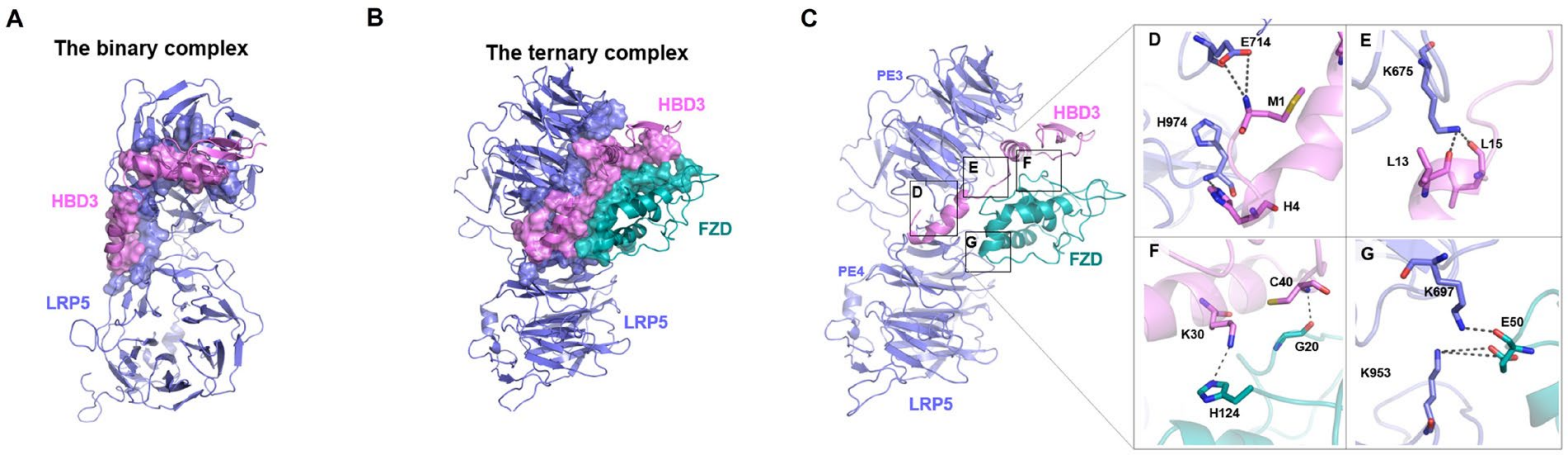
The plausible binding modes and critical interactions of the binary (LRP5-HBD3) and ternary (LRP5-HBD3-FZD8) complexes. **(A,B)** Protein–protein docking and molecular dynamics simulations revealed potential binding modes of the LRP5-HBD3 and LRP5-HBD3-FZD8 interaction complexes, respectively. The residues involved in the formation of the complex are shown in surface representation, and LPR5, HBD3, and FZD8 are colored in steel blue, light pink, and teal, respectively. HBD3 binds between the β-propeller domain-3 of LPR5 and the cysteine-rich domain of the FZD8 receptor. FZD8 receptor interacts with HBD3 as well as with the LRP5 receptor residues. **(C)** Stable H-bond interactions observed during the MD simulations of the complex are highlighted in boxes. **(D)** The sidechain carboxylic group of E714 of LRP5 interacts with the backbone amide nitrogen of M1 of HBD3, and the backbone amide nitrogen of H974 of LRP5 interacts with side chain ε nitrogen of H4 of HBD3. **(E)** The sidechain amino group of K675 of LRP5 interacts with backbone carbonyl oxygens of L13 and L15 of HBD3. **(F)** The sidechain amino group of K30 of HBD3 interacts with the ε nitrogen of FZD8 H124. Similarly, the backbone amide nitrogen of C40 of HBD3 forms an H-bond with the backbone carbonyl oxygen of FZD8 G20. **(G)** The sidechain terminal amino group of K953 of LRP5 forms a salt bridge with the sidechain carboxyl group of E50. Similarly, the sidechain amino group of K697 of LRP5 formed an H-bond with the backbone carbonyl oxygen of E50 of the FZD8 receptor.

LRP5 belongs to a single-pass transmembrane family of proteins. The extracellular portion of LRP5 consists of four β-propeller domains (P1, P2, P3, P4) connected by epidermal growth factor (EGF)-like domains (E1, E2, E3, E4), and three LDLR (low-density lipoprotein receptor) type A (LA) domains preceding the transmembrane helix (Figure 7B; Supplementary Figures S2A,B; Matoba et al., 2017). Each pair of the four β-propeller and EGF-like domains are designated as PE1, PE2, PE3, and PE4 (Figure 7B). As the LRP5 structure is unavailable, LRP6, the nearest homolog with ~70% sequence identity, was used as a template for homology modeling of the PE3-PE4 domains of LRP5. Earlier studies indicate that Wnt and other ligands of LRP5 bind at the PE3 domain of LRP5 (Ahn et al., 2011; Bourhis et al., 2011; Cheng et al., 2011; Zebisch et al., 2016; Hirai et al., 2019). Therefore, the residues from the LRP5-PE3 domain namely, A667, V694, E721, T737, N762, W780, R805, D824, H847, W863, V889, and M890 were chosen as the interaction site for docking of HBD3 (Supplementary Figure S2C; Cheng et al., 2011). Since there were no sequence similarities between HBD3 and other known LRP5 interacting proteins, all residues of HBD3 were considered for docking. As a novel binding partner of LRP5, the docking of HBD3 without predefined residues was expected to predict the potential binding interface between LRP5 and HBD3 in an unbiased manner. The optimal binding conformation of HBD3 with LRP5 with a HADDOCK score of −146.2 showed several non-bonded interactions at the interface. HBD3 residues such as Y5, P19, G20, I24, G23, T27, R31, Y32, and R36 formed prominent interactions with the LRP5 PE3 domain residues R652, E676, S695, Y719, W780, R805, D824, H847, H974 and G1007 to form a stable complex. The resultant HBD3-LRP5 complex was then used for subsequent docking to the FZD8 receptor (Figure 8A).

#### Docking of FZD8 to the LRP5-HBD3 complex

FZD8 receptors are class F G protein-coupled receptors (GPCRs) and are known to activate the canonical β-catenin pathway (Wright et al., 2019). FZD8 receptors form heterodimeric signaling complexes with LPR5/6 through their conserved extracellular cysteine-rich domain (Tsutsumi et al., 2020). The FZD8-Wnt complex’s crystal structures show that Wnt interacts with FZD8 at two locations to form the complex (Supplementary Figure S3A). Therefore, the LPR5-HBD3 complex was docked independently at the two known sites of the FZD8 receptor, namely site-1 and site-2. Site-1 comprised of residues, such as E68, Q71, F72, Y92, F127, P130, D131, and R132 and site-2 comprised of residues, such as I46, Y48, F86, I95, L97, D99, Y100, K102, L104, L147, M149, D150, and N152 (Supplementary Figures S3B,C). The HBD3 residues, except those interacting with LRP5, were chosen as the potential FZD8-interacting site. Among the two sites of the FZD8 receptor, site 2 exhibited a higher binding affinity towards the LRP5-HBD3 complex. The binding energy, calculated as docking score, of the FZD8 with LRP5-HBD3 complex at site 2 (−95.7) was observed to be higher than that of site 1 (−85.3). In this high-affinity binding mode, FZD8 formed interactions with both HBD3 and the LRP5 receptor. Most notably, in this binding mode, HBD3 was seen sandwiched between FZD8 and LRP5, reminiscent of the LRP5-Wnt-FZD8 complex (Figures 8A,B). Several residues from site 2 of FZD8, including I46, G47, E77, P82, D83, F86, E98, D99, Y125, and Y151, formed non-bonded contacts with the residues of HBD3 (C40, V42, K48, R58, R60, and R65) and LRP5 (K953, R997, and H1197), respectively (Figures 8C–G).

#### MD simulations of the binary and ternary complexes

To examine the stability of the interactions among the LRP5-HBD3 and LRP5-FZD8-HBD3 complexes, we performed MD simulations of the complexes, each for 500 ns. Overall, the binary and ternary complexes were observed to be stable throughout the simulation time. During the first 50–100 ns of the simulations, the complexes underwent notable conformational changes (reflected by the RMSD value of ~5 Å), finetuning interactions between the interfaces, and then stabilized through the rest of the simulation time (Supplementary Figure S4).

#### H-bonds at the interfaces

**Figure 9:**
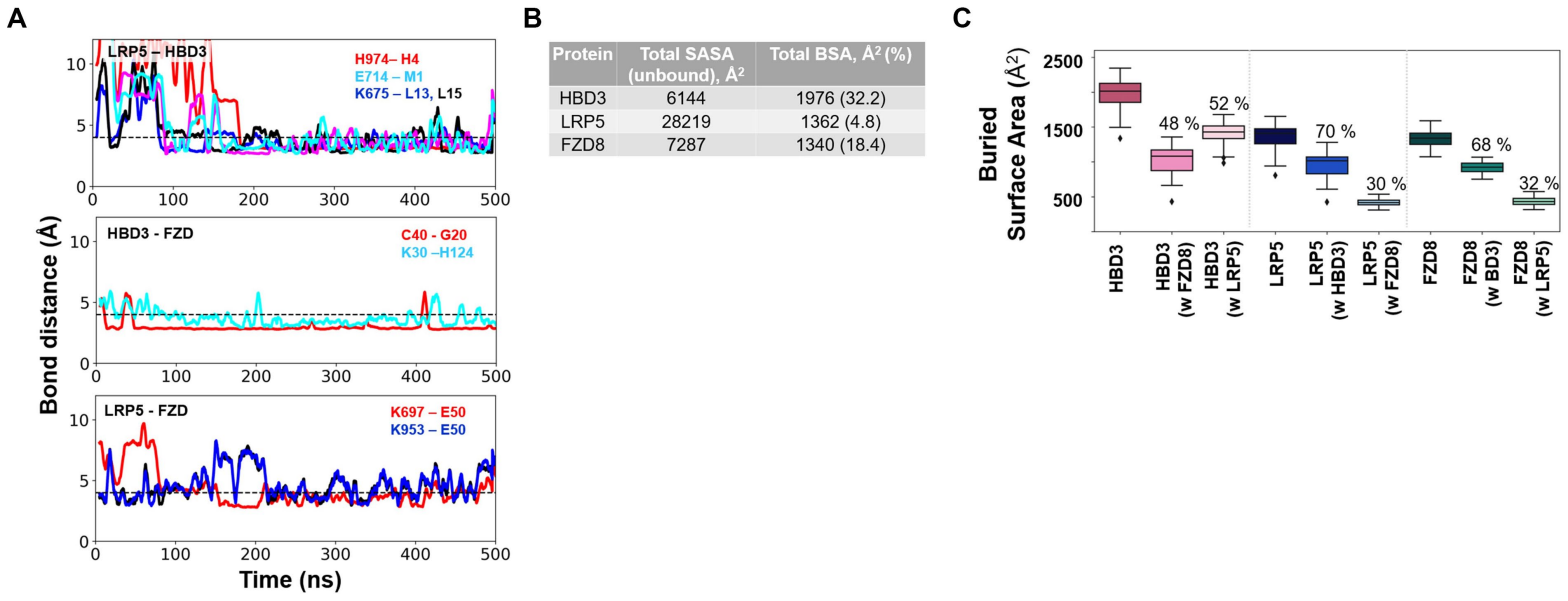
Critical H-bond interactions and the extent of protein–protein interactions as measured by the buried solvent-accessible surface area (SASA) of individual proteins upon complex formation. **(A)** Multiple stable H-bond interactions were observed in the LRP5-HBD3-FZD8 ternary complex. H-bond interactions between LRP5-HBD3, HBD3-FZD8, and LRP5-FZD8 are shown. **(B)** Total solvent-accessible surface area (SASA) of the three proteins in their unbound states and the amount of SASA lost due to complex formation, called buried surface area (BSA) and %, are given. **(C)** The total BSA and the fraction of the buried surface area for each interacting partner of HBD3, LRP5, and FZD8 receptors were calculated from the MD simulation. The BSA of HBD3 shows that HBD3 shares its interaction surface approximately equally with LRP5 and FZD8 receptors. On the other hand, LRP5 and FZD receptors share a larger fraction of interaction surface with HBD3. The fraction of interaction surface for LRP5 and FZD8 receptors was relatively smaller in comparison to their total SASA.

To evaluate the critical H-bonds involved in formation and stabilization of the complexes, all three interface hydrogen bond interactions (LRP5-HBD3, HBD3-FZD, and FZD8-LRP5) were analyzed (Figures 8A,B). Several H-bonds were observed among the interacting proteins. However, only a set of H-bonds were found stable for most of the simulation time and are discussed here (boxes in Figure 8C). Interestingly, there were many H-bonds observed in the LRP5-HBD3 interface, of which the most stable ones formed around 100 ns into the simulation. The N-terminal residues M1 and H4 of HBD3 were found to interact with sidechain carboxylic oxygens of E714 and backbone amide nitrogen of H974 of LRP5, respectively (Figure 8D). During the simulation, M1 and H4 of HBD3 moved closer to E714 and H974 of LRP5 and formed H-bonds. Similarly, the sidechain amino group of K675 forms H-bonds with backbone carbonyl oxygens of L13 and L15 of HBD3 (Figure 8E). These H-bonds with were found to be consistent after 200 ns of the simulation (Figure 9A).

In the case of the HBD3-FZD8 interaction interface, K30 of HBD3 formed H-bonds with sidechain ε nitrogen of H124 (Figure 8F). Similarly, the C40 backbone amide nitrogen of HBD3 interacted with the backbone carbonyl oxygen of G20 of FZD8. All these H-bonds were observed to be flexible during the initial 80 ns but gradually stabilized for the rest of the simulation time (Figure 9A). In the FZD8-LRP5 interaction interface, two lysine residues, K697 and K953, from PE3 and PE4 β-propeller domains of LPR5, were interacting with E50 of FZD8. The sidechain amino group of K697 from the PE3 β-propeller domain of LRP5 forms an H-bond with the backbone carbonyl oxygen of E50 of FZD8. A salt bridge interaction between the sidechain amino group of K953 (PE4 β-propeller of LRP5) and the carboxyl group of E50 (FZD8) was found to be stable at the FZD8-LRP5 interaction interface (Figure 8G). The FZD8 receptor interacts and forms stable H-bonds with both HBD3 and LRP5 receptors. A comparison of the H-bonds between FZD8-HBD3 and FZD8-LRP5 showed that the polar contacts are becoming stronger after the initial fine-tuning of the interaction surfaces (Figure 9A). Overall, the variation and flexibility in interactions illustrate the adaptability of the interaction surfaces between the receptors.

#### Hydrophobic interactions quantified as percent contact occupancy

To evaluate the critical interactions involved in stabilizing the complex, residue contacts at all three interfaces of complexes (LRP5-HBD3, HBD3-FZD8, and FZD8-LRP5) were analyzed. We applied a distance-based cutoff (4 Å) to evaluate all non-bonded interactions involved in the interacting surfaces. As several residue interactions were observed among the interacting proteins, only interactions that were observed in more than 80% of the simulation time were discussed here. From the contact analysis of the LRP5-HBD3 interface, the residues G673, K675, E676, H711, E714, F715, W780, R805, H847, F849, W863, F888, M890, P972, L973, H974, G975 and P1010 of LRP5 were observed to be in contact with the HBD3 residues, including M1, R2, H4, Y5, L13, I24, I25, L28, Y31, Y32 and V35. The N-terminal residue M1 of HBD3 forms contact with the PE3 β-propeller domain in the cleft formed in between the PE3 and PE4 β-propeller domains. In addition, the α helix in HBD3, formed by residues 24–36, mainly interacted at the surface of the PE3 domain (Figure 8A; Supplementary Figure S5).

HBD3 was sandwiched between LRP5 and FZD8. HBD3 residues, such as F8, L11, F12, L15, P17, V18, K30, R34, G37, R39, and C40, formed contacts with the FZD8 receptor residues, including G18, G20, Y21, Q22, I51, Q52, C53, S54, P55, P93, L94, Q97, and Y98. Interestingly, HBD3 appears to engage in a significantly greater number of contacts with FDZ8 than with LPR5 (Figure 8C; Supplementary Figure S6). In our molecular docking studies, the interaction of the FZD8 receptor with LRP5 was unexpected and different from the previously described LRP5-Wnt-FZD8 interaction model. Interestingly, in addition to the interactions with HBD3, the FZD8 receptor also had contact with the LRP5 receptor residues throughout the simulation period. Specifically, the residues of FZD8, W46, P47, E50, Q52 and Y98 were in contact with the S695 and K697 of the PE3 β-propeller domain and K953, S954, R977, A1196 and H1197 of the PE4 β-propeller domain of LRP5 (Figure 8G; Supplementary Figure S7).

#### The extent of protein–protein interactions was quantified as the buried solvent-accessible surface area upon complex formation.

We performed the solvent-accessible surface area calculation (SASA) to estimate the extent to which the proteins’ surfaces were buried (buried surface area, BSA) during the formation of LPR5-HBD3 and LPR5-HBD3-FZD3 complexes. The SASA values represent the average calculated through the entire 500 ns MD simulations. The fraction of BSA for each protein is significantly different, ranging from 5 to 30% (Figure 9B). The PE3-PE4 domains of LPR5 are much larger than HBD3 and the CRD domain of FZD8. Only ~4.8% of the LPR5 PE3-PE4 domain’s surface was buried in the complex formation, of which ~70% of the surface was engaged in interactions with HBD3 and ~ 30% of the surface was involved in interactions with FZD8 (Figure 9C). Similarly, ~32.17% of the surface of HBD3 was buried in the complex, of which ~52% of the surface involved in the interactions with LPR5 and ~48% with FZD8 receptor, which indicates that HBD3 shares the interaction surface almost equally with LRP5 and FZD8 receptors (Figure 9C). In the case of FZD8 receptor CRD, ~18.39% of the surface was buried in the complex, of which ~68% of the surface was engaged in the interactions with HBD3 and ~32% with LRP5 (Figure 9C). In addition, we have calculated the polar and non-polar fractions of the total SASA, which revealed that the ternary complex is stabilized mostly by non-polar interactions (Supplementary Figure S8). From the comparison of the BSA of the LPR5, HBD3, and FZD8, it was clear that HBD3 is sandwiched between LRP5 and FZD8 receptors and interacts through distinct surfaces. Moreover, both LRP5 and FZD receptors share larger fractions of their interaction surfaces with HBD3, indicating stable and favorable interactions.

## Discussion

There is an urgent need to develop antiviral therapeutics and vaccines for Respiratory Syncytial Virus (RSV) since it is a leading cause of mortality and morbidity among infants, the elderly, and immunocompromised patients (Diseases, 1993; Falsey et al., 2005; Nair et al., 2010; Hurwitz, 2011; Graham and Anderson, 2013; Griffiths et al., 2017; Romero et al., 2017). To achieve this goal, it is critical to understand RSV-host interactions and mechanisms that trigger the pro-inflammatory response. Pro-inflammatory response plays a critical role in developing severe airway inflammatory diseases such as pneumonia and bronchiolitis during RSV infection (Imai et al., 2008; Murawski et al., 2009; Ruuskanen et al., 2011; Foronjy et al., 2016; Russell et al., 2017). Additionally, inflammatory response constitutes a key cellular immune mechanism dictating vaccine efficacy. In the current study, we have identified a yet unknown pro-inflammatory pathway utilized by the host during RSV infection. We show the essential role of β-catenin dependent signaling pathway in positively regulating pro-inflammatory response in RSV-infected human lung epithelial cells.

The canonical Wnt/β-catenin pathway is a key signal transduction pathway involved in a wide spectrum of biological mechanisms (Clevers, 2006; Zhan et al., 2017; Ren et al., 2021; Rim et al., 2022), including regulating immunity (Ma and Hottiger, 2016). One specific mechanism comprises of expressing pro-inflammatory genes that culminate in inflammation. β-catenin signaling pathway promoted expression and production of pro-inflammatory cytokines/chemokines from macrophages (Zhao et al., 2015; Huang et al., 2018). Towards that end, the β-catenin signaling pathway also contributed to lung inflammation during sepsis (Sharma et al., 2017). Compared to macrophages, the role of β-catenin in epithelial cells during pro-inflammatory response is very limited. Only one study reported the involvement of β-catenin in stimulating pro-inflammatory cytokines in LPS-treated bronchial epithelial cells (Jang et al., 2014). Studies focusing on the role of β-catenin during infection with RNA respiratory viruses are limited. A study showed Wnt/β-catenin signaling pathway regulating influenza A virus (IAV is an orthomyxovirus) replication in epithelial cells (More et al., 2018). Additionally, we have previously demonstrated the trans-activation of the human parainfluenza virus type 3 gene by β-catenin (Bose and Banerjee, 2003). However, the role of β-catenin as a pro-inflammatory mediator during infection with RNA respiratory viruses is limited. Especially, it was unknown whether pneumoviruses like RSV trigger β-catenin activation and the role of such activation during virus-host interaction. In the current study, we demonstrated β-catenin activation by RSV and further showed the role of β-catenin in positively regulating pro-inflammatory response by virtue of inducing the production of key pro-inflammatory chemokine IL-8. In contrast to earlier studies with IAV, β-catenin signaling had no role in RSV replication/infection, but it was involved in the production of pro-inflammatory mediator IL-8.

Lung injury during RSV infection is associated with an influx of inflammatory immune cells into the airway (Imai et al., 2008; Murawski et al., 2009; Ruuskanen et al., 2011; Foronjy et al., 2016; Russell et al., 2017). Neutrophils constitute one of the infiltrating inflammatory cell types contributing to exaggerated lung inflammation and injury during RSV infection (Everard et al., 1994; McNamara et al., 2003; Emboriadou et al., 2007; Sebina and Phipps, 2020). Neutrophil recruitment to the RSV-infected lower respiratory tract is mediated by chemokine IL-8 released from lung epithelial cells and monocytes (Baggiolini et al., 1989; Bazzoni et al., 1991; Biswas et al., 1995; Bont et al., 1999). IL-8 (encoded by the CXCL8 gene in humans) is a chemokine with a C-X-C motif that acts as a potent chemoattractant for neutrophils (Baggiolini et al., 1989; Bazzoni et al., 1991). High levels of IL-8 in respiratory secretions have been detected in RSV-infected infants with severe respiratory disease (Biswas et al., 1995). IL-8 also serves as a biomarker defining lung disease severity in RSV-infected infants (Bont et al., 1999). IL-8 is the major chemokine secreted by RSV-infected lung epithelial cells, including human lung epithelial A549 cells (Fiedler et al., 1995; Thomas et al., 2000; Rudd et al., 2005). Interestingly, in A549 cells, IL-8 constitutes the only pro-inflammatory mediator detected at appreciable levels compared to other pro-inflammatory factors like TNF, IL-6, and IL-1β. IL-8 production during RSV infection is triggered by two transcription factors, NF-kB and NF-IL6 (Jamaluddin et al., 1996; Mastronarde et al., 1996). Interestingly, a study with human hepatocytes has identified the IL-8 gene as a direct target of β-catenin and TCF4 transcription factors (Lévy et al., 2002). A consensus TCF/LEF site was detected in the IL-8 promoter, and that site was essential for the transactivation of the IL-8 gene by β-catenin in association with the p300 co-activator (Lévy et al., 2002). Moreover, the β-catenin pathway was involved in positively regulating the expression and release of IL-8 from macrophages (Masckauchan, 2005) and endothelial cells (Jang et al., 2014) following allergic reactions and angiogenesis, respectively. However, the role of β-catenin, if any, in IL-8 production during virus infection has not been investigated yet. Our current study has highlighted the involvement of β-catenin signaling in triggering IL-8 production during RSV infection. Based on the critical role of IL-8 in conferring exaggerated airway inflammation during RSV infection (Baggiolini et al., 1989; Bazzoni et al., 1991; Everard et al., 1994; Biswas et al., 1995; Bont et al., 1999; McNamara et al., 2003; Emboriadou et al., 2007; Sebina and Phipps, 2020), our results have unfolded a new virus-host mechanism required for the efficient production of potent chemotactic factors like IL-8.

In addition to unfolding the β-catenin pathway as an IL-8 inducer during RSV infection, we have also identified a new mechanism triggering β-catenin activation during viral infection. We have identified human beta-defensin 3 (HBD3) as an extracellular ligand involved in activation β-catenin by virtue of interacting with the well-established Wnt receptor LRP5 which is part of the β-catenin activating Fzd-LRP5/6 receptor complex. Defensins are anti-microbial cationic peptides regulating pathogen burden, immunity, and host defense (Fruitwala et al., 2019; Xu and Lu, 2020). Apart from the anti-microbial property, we and others have demonstrated the additional role of defensins in controlling virus infection. Specifically, beta-defensins are involved in counteracting virus infection by various mechanisms. We have previously identified HBD2 and HBD3 as key epithelial cell-derived beta-defensins involved in controlling RSV and VSV (Vesicular Stomatitis Virus is a non-segmented negative-sense RNA virus like RSV) infections by blocking viral cellular entry (Kota et al., 2008; Basu et al., 2010). However, the role of beta-defensins during cellular signaling of RSV-infected cells has not been reported previously. Particularly, it was unknown whether beta-defensins can modulate pro-inflammatory response in RSV-infected cells. Based on a previous study showing β-catenin activation by HBD3 in human non-epithelial dental cells (human periodontal ligament cells; Zhou et al., 2018), we investigated whether HBD3 can act as a non-canonical ligand (i.e., compared to canonical Wnt ligand) for β-catenin activation in RSV infected lung epithelial cells. Our study demonstrated that HBD3 released from RSV-infected cells is involved in activating β-catenin during RSV infection. Furthermore, our interaction studies showed direct interaction of HBD3 with LRP5, a receptor that is part of the multi-protein receptor complex (Fzd-LRP5/6) involved in Wnt-mediated β-catenin activation.

HBD3 interacts with at least three cell surface receptors, melanocortin receptors, CCR6, and CD98 (Semple et al., 2010; Colavita et al., 2015; Shelley et al., 2020). However, none of these receptors are involved in immune signaling. Our study has identified LRP5 as a new HBD3 receptor involved in pro-inflammatory signaling. Moreover, we show that HBD3-LRP5 interaction may drive activation of the β-catenin pathway leading to a pro-inflammatory response due to the production of potent neutrophilic chemokine IL-8. HBD3 interacted with cell surface localized LRP5, and such interaction was mediated via direct protein–protein interaction. The extracellular domain of LRP5 is comprised of – (a) four β-propeller domains, (b) four EGF-like domains, and (c) one LDLR type A domain (Clevers, 2006; Zhan et al., 2017; Ren et al., 2021; Rim et al., 2022; Figure 7B). The four β-propeller and four EGF-like domains are localized tandemly and designated as PE1, PE2, PE3, and PE4. During canonical Wnt/ β-catenin signaling, the canonical ligand Wnt binds to these domains to activate β-catenin signaling. To date, 10 Wnt ligands exist, and previous studies have shown that Wnt3a is involved in β-catenin activation in IAV-infected lung epithelial cells (More et al., 2018). Wnt3a binds to the PE3 domain of the LRP5 protein. Interestingly, purified HBD3 is also bound to the PE3 domain of LRP5 protein, as deduced from our *in vitro* interaction studies with purified HBD3 and truncated LRP5 proteins. Our in-silico studies also provided a structural basis for the interactions between LRP5 and HBD3. Importantly, we obtained the potential binding orientations and critical residues at the interacting surfaces of the proteins using protein–protein docking. Subsequently, we evaluated the stability of the complex using 0.5 μs long MD simulations (Figure 8). Our simulations revealed that HBD3 interacts with LRP5 at its PE3 propeller domain, similar to other Wnt ligands reported before. The polar H-bond and nonpolar hydrophobic interactions between the two protein surfaces were assessed qualitatively by monitoring the H-bond distances and contact occupancy (%), respectively. Multiple H-bonds were observed between the LRP5-HBD3 interface. Similarly, the contact occupancy provides the fraction of the simulation time during which residues from the two proteins are within 4 Å distance (Figure 9; Supplementary Figure S5), indicating van der Waal and hydrophobic interactions. Both parameters indicate stable and lasting interactions between the proteins, involving the burial of greater than 30% of the solvent-accessible surface area of HBD3.

**Figure 10:**
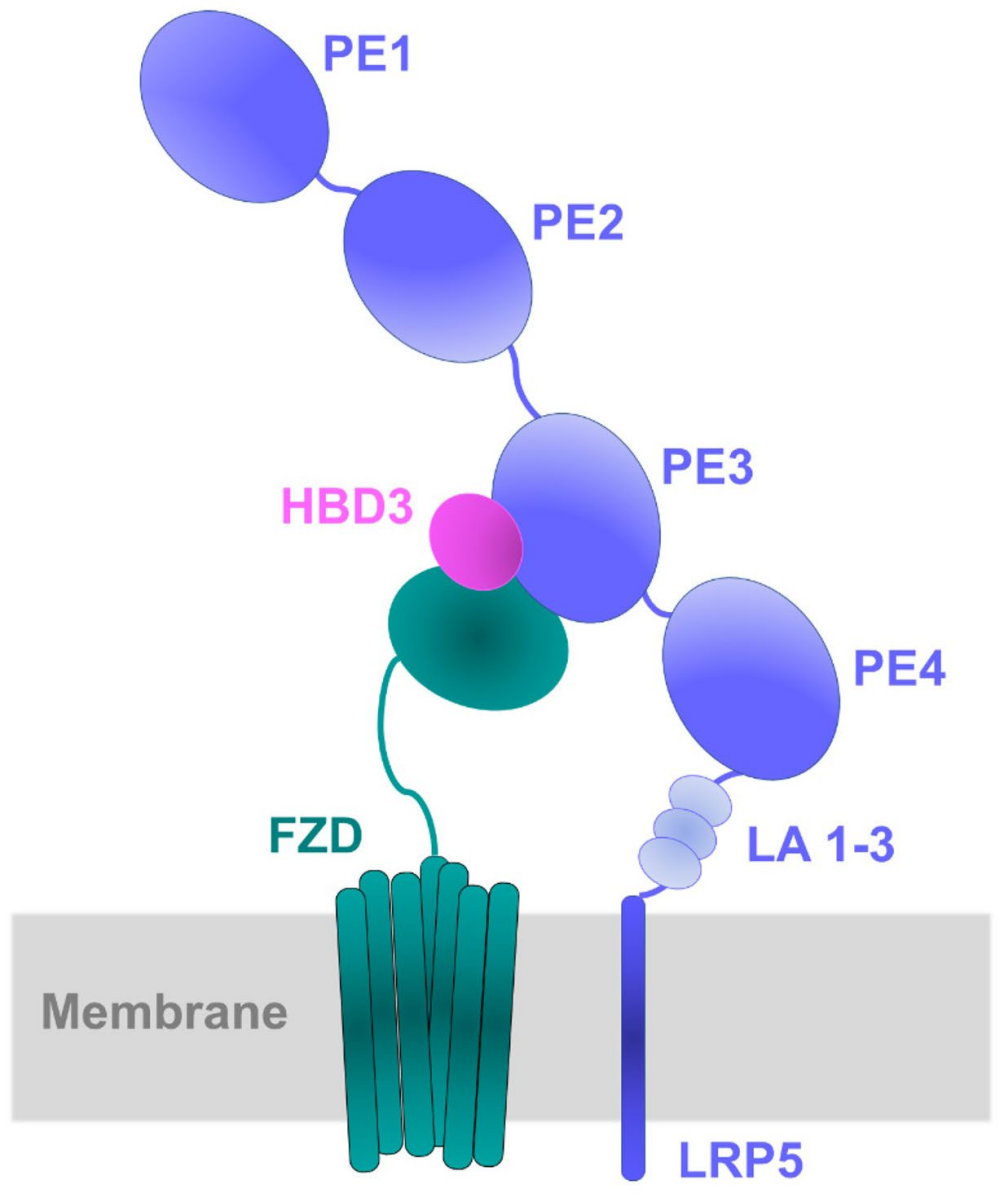
Schematic diagram of the proposed model for LRP5-HBD3-FZD8 ternary complex. Protein–protein docking, and MD simulations predicted the potential interactions of HBD3 with LRP5 and FZD8. HBD3 appears to be sandwiched between LRP5 and FZD8. Specifically, HBD3 binds mostly to the PE3 propeller domain of LRP5 protein.

Earlier studies have shown that the Wnt ligands act at the cell surface by forming a heterotrimeric ternary complex with LRP5 and frizzled receptors such as FZD8 (Hirai et al., 2019). We modeled such a ternary complex of HBD3 with LRP5 and FZD8 in which HBD3 was sandwiched between the two other proteins (Figure 10). The extracellular cysteine-rich domain of FZD8 was successfully docked to the HBD3 surface distal to its LRP5-binding interface. Surprisingly, the 0.5 μs-long MD simulations of this ternary complex revealed unexpected interactions of FZD8 with both HBD3 and the upper region of the PE4 domain of LRP5. These interactions were quite stable and lasted for the entire simulation time. Similar to the binary LRP5-HBD3 complex, both polar H-bond distances and contact occupancy (%) indicated the stability of the residue interactions of the ternary complex. Based on the observations, we propose this ternary model of LRP5-HBD3-FZD8 as potential mechanism through which HBD3 produces a proinflammatory response via the β-catenin pathway (Figure 10).

In summary, our studies have identified the HBD3/β-catenin signaling pathway as a new non-canonical β-catenin activating pathway involved in triggering a pro-inflammatory response in RSV-infected lung epithelial cells. We envision that the HBD3/β-catenin network acts both via paracrine and autocrine loop to amplify the inflammatory response in non-infected and infected cells, respectively.

## Materials and methods

### Cell culture and viruses

Human lung epithelial cells (A549) were purchased from American Type Culture Collection (ATCC). Cells were cultured in complete Dulbecco’s modified Eagle medium (DMEM; Gibco) containing 10% fetal bovine serum (FBS), 100 IU/ml penicillin, and 100ug/ml streptomycin unless otherwise stated (Kota et al., 2008; Chang et al., 2012; Mgbemena et al., 2012, 2013). Human respiratory syncytial virus (RSV A2 strain) was purified as described previously (Kota et al., 2008; Chang et al., 2012; Mgbemena et al., 2013). Recombinant human RSV expressing mKate2 protein (mKate2-RSV) was propagated from pSynk-A2 as described previously (Hotard et al., 2012; Meng et al., 2014; Bedient et al., 2020). pSynk-A2 and helper plasmids were provided by Dr. Martin Moore (Emory University) and BSRT7/5 cells were provided by Dr. Ursula Buchholz (National Institutes of Health).

### Cell treatment and virus infection

Cells were infected with RSV and mKate2-RSV at the multiplicity of infection (MOI) of 1 or 3. Virus adsorption was performed for 1.5 h (at 37°C) in OPTI-MEM medium (Gibco). Following adsorption, cells were washed with Dulbecco’s phosphate-buffered saline (DPBS; Gibco) and infection was continued in the presence or absence of a complete medium. In some experiments, cells were pre-treated with either vehicle (DMSO) or Wnt/β-catenin pathway inhibitors (iCRT3 or iCRT14; 25uM; Sigma) and infected with RSV in the absence and presence of these inhibitors. For human beta-defensins 3 (HBD3) treatment studies, A549 cells were treated with 10ug/ml of purified HBD3 (Peprotech) or vehicle (PBS with 0.1% BSA). Cells were also treated with either lithium chloride (LiCl; Sigma) or vehicle (water) for 24 h. A549 cells were transfected with either empty-FLAG plasmid or FLAG-tagged LRP5 (FLAG-LRP5; 200 ng/ml) by using Lipofectamine 2000 (Life Technologies).

### Generation of A549 cells expressing truncated β-catenin protein

A549 cells expressing either full-length β-catenin protein (FL-catenin) or truncated β-catenin protein (Δ-catenin) were generated by Synthego Corporation using CRISPR-Cas9 technology with the sgRNA sequence of GAGTGGTAAAGGCAATCCTG located in exon 3. Individual clones were isolated by limiting dilution from the cell pool provided by Synthego. To identify those that would likely have diminished activity without being a null, the mutation(s) carried by each clone was determined by PCR amplification of the region surrounding the sgRNA target site using F primer 5′ATCCCCCTGCTTTCCTCTCT3′ and R primer 5′ACATAGCAGCTCGTACCCTC3′. Clones that carried deletions were sequenced to determine the mutation.

### Luciferase assay

Cells (A549, FL-catenin, and Δ-catenin cells) were co-transfected with M50 Super 8× TOP-Flash firefly luciferase reporter plasmid (Addgene) and a plasmid encoding Renilla luciferase for 16 h. Transfected cells were either infected with RSV or treated with purified HBD3 protein. Luciferase plasmid-transfected FL-catenin and Δ-catenin cells were also treated with LiCl. Following infection/treatment, the Dual-Luciferase® reporter assay system (Promega) was used for luciferase analysis. Luciferase activity was determined using a microplate luminometer (Promega).

### siRNA transfection

Scrambled control siRNA and HBD3 siRNA were purchased from Santa Cruz Biotechnology. A549 cells were transfected with these siRNAs (60 pmol) by using Lipofectamine 2000 (Life Technologies). HBD3 silencing was confirmed by western blotting with an HBD3 antibody.

### ELISA

Human IL-8 ELISA kit was purchased from Invitrogen. HBD3 ELISA kit was obtained from MyBioSource. IL-8 and HBD3 levels in the medium supernatant were determined following the manufacturer’s instructions. ELISA values of the experimental group (i.e., RSV-infected cells or cells infected with RSV in the presence of iCRT3 or iCRT14) represent values obtained following subtraction of background signal from the control group (i.e., mock cells or cells infected with RSV in the presence of control vehicle).

### Western blot

A549 cells were lysed using 1%-Triton X-100 (pH 7.4), EDTA-free protease inhibitor cocktail (Roche Diagnostics) in PBS. Cell lysates were subjected to SDS-PAGE and separated proteins were transferred onto 0.2 μm nitrocellulose membrane (GE Health care) and blotted with specific antibodies. β-catenin and FLAG antibodies were purchased from Sigma-Aldrich. LRP5 antibody was obtained from Cell signaling. β-actin antibody was purchased from Bethyl Laboratories. HBD3-8A antibody was deposited to the DSHB by Starner, T. (DSHB Hybridoma Product hBD-3-8A). An anti-RFP antibody was purchased from Invitrogen. Western blots were quantified using ChemiDoc™ XRS + software Image Lab 5.1 (BioRad).

### RNA isolation and reverse transcriptase-PCR (RT-PCR)

Total RNA was extracted using TRIzol reagent (Life Technologies) following supplier’s instructions. Isolated RNA was treated with RNase-free DNase I (Thermoscientific) and cDNA was synthesized using a High-Capacity cDNA Reverse Transcription Kit (Applied Biosystems). RT-PCR was performed using 2X Taq Red master mix (Apex) in a final reaction of 25 μl. The amplification cycle was as follows: an initial denaturing step (95°C for 3 min) was followed by 34 cycles of denaturing (95°C for 30 s), annealing (55.7°C for 30 s–), and extending (72°C for 1 min), followed by 5 min at 72°C for elongation. Following amplification, the PCR products were analyzed on 2% agarose gels and bands were visualized by ChemiDoc XRS (BioRad). The PCR product bands were quantified using ChemiDoc™ XRS + software Image Lab 5.1 (BioRad). Housekeeping gene glyceraldehyde-3-phosphate dehydrogenase (GAPDH) was used as a loading control. The primers used to detect the indicated genes are listed below:

Human GAPDH forward, (5′ GATCATCAGCAATGCCTCCT-3′) and human GAPDH reverse, (5′ TGTGGTCATGAGTCCTTCCA-3′).

Human HBD3 forward, (5′ TCCAGGTCATGGAGGAATCAT-3′) and human HBD3 reverse, (5′ CGAGCACTTGCCGATCTGT-3′).

### Interaction of biotinylated HBD3 protein with LRP5 protein

The EZ-link® TFPA-PEG3-Biotin kit (Thermo Fisher Scientific, Massachusetts, USA) was used to biotinylate purified HBD3 protein (Peprotech). Biotinylation was performed in the dark as per the manufacturer’s instructions. For cell surface interaction studies, A549 cells were transfected with either an empty-FLAG vector or LRP5-FLAG for 16 h. After 16 h, cells were cooled to 4°C for 2 h, and the chilled cells were subsequently incubated with biotinylated-HBD3 for 4 h at 4°C. Cells were lysed with PBS containing 1% TritonX-100 and protease inhibitors. Cell lysates were incubated with NeutrAvidin-agarose beads (Thermo Fisher Scientific) for 16 h at 4°C. After washing the agarose beads with wash buffer (10 mM Tris–HCL with protease inhibitor), the proteins bound to avidin–agarose beads were subjected to western blotting with anti-FLAG antibody. For cell-free interaction studies, we biotinylated purified HBD3 protein and purchased purified truncated LRP5 protein with Fc tag (purchased from antibodies online.com). The purified truncated LRP5 protein consisted of E3 and E4 regions (aa769–aa1016) of the LRP5 extracellular domain. For the interaction studies, biotinylated-HBD3 and non-biotinylated HBD3 (control) were incubated (16 h at 4°C) with avidin–agarose beads. Following washing, the agarose beads were further incubated (16 h at 4°C) with purified truncated LRP5 protein. Washed beads were boiled with SDS sample buffer, followed by SDS-PAGE and western blotting with LRP5 antibody.

### Statistical analysis

All data were analyzed using GraphPad Prism software (6.0). For ELISA and luciferase assay, a significance test was carried out using Student’s *t*-test. Western blot densitometric values were quantified by using ChemiDoc™ XRS + software Image Lab 5.1 (BioRad), and Student’s *t*-test was utilized to determine significance.

### Modeling of LRP5-HBD3-FZD complex structure

#### Structures

To examine the potential interactions of HBD3 with LRP5 and Frizzled-8 receptor (FZD8), we assembled the binary (HBD3-LRP5), and ternary (HBD3-LRP5-FZD8) complexes using protein–protein docking of the three proteins. Multiple experimental structures are available for FZD8, whereas the structures for LRP5 and HBD3 are yet to be resolved. For FZD8, we used the X-ray crystal structure of the protein in complex with a surrogate Wnt agonist (PDB ID: 5UN5, resolution of 2.99 Å; Janda et al., 2017). The structure of LRP5 was modeled using the X-ray crystal structure of its closest homolog, LRP6 (PDB ID: 4DG6, resolution 2.9 Å), using modeler v10.3 (Webb and Sali, 2016). From the generated model structures, the structure with the least energy refinement score was considered the best model (Shen and Sali, 2006). Similarly, for HBD3, only a partial NMR structure is available in the PDB database. Hence the full-length model from the AlphaFold database is used (https://alphafold.ebi.ac.uk/entry/P81534; Varadi et al., 2022). The quality of the generated models (LRP5 homology model and HBD3 Alpha fold model) was further validated by the Ramachandran plot using the SAVES server (https://saves.mbi.ucla.edu/; Bowie et al., 1991; Lüthy et al., 1992; Colovos and Yeates, 1993; Pontius et al., 1996). The details are provided as Supplementary Figures S9, S10. Finally, the modeled structures were further geometry-optimized to remove any bad contacts and energy-minimized using MOE v2015 and used for subsequent protein–protein docking and MD simulations (Ccgi, 2016).

#### Protein–protein docking

For modeling the binary and ternary complexes of LRP5, FZD8, and HBD3, sequential protein–protein docking simulations were carried out in two steps – (1) docking of HBD3 with LPR5, and (2) docking of the LRP5-HBD3 complex with FZD8. For docking of HBD3 with LRP5, the following residues of the PE3 (β propeller) domain of LRP5 (A667, V694, E721, T737, N762, W780, R805, D824, H847, W863, V889, and M890) were selected as the potential interaction site (Supplementary Figures S2C). For HBD3, all residues were selected to generate all possible binding conformations of HBD3 with LPR5. Furthermore, to generate a ternary FZD8-LRP5-HBD3 complex, the LRP5-HBD3 complex obtained from the previous step was docked to FZD8, using the residues of HBD3 that do not interact with LRP5, as the potential interaction site. In the case of FZD8, two possible binding interfaces were selected and docked individually. All the protein–protein docking simulations were performed using the HADDOCK v2.4 server (https://bianca.science.uu.nl/haddock2.4; Honorato et al., 2021). For each protein–protein docking simulation, at most 30,000 possible binding orientations were generated, amongst which 2000 poses were considered for post-docking minimization, and finally, 1,000 poses were filtered and used for scoring and clustering. Apart from these, other docking parameters were kept at their default values in HADDOCK. Among the resulting clusters with multiple docking poses, a cluster with plausible binding orientations and interactions and the lowest binding energies was considered for subsequent MD simulations.

#### MD simulations

To examine the stability, binding modes, and residue interactions of the binary and ternary complexes, we performed unbiased MD simulations using GROMACS v2021 (Abraham et al., 2015). The input files for the MD simulations were generated using the Input Generator-Solution Builder module of CHARMM-GUI (Lee et al., 2016) with CHARMM36 forcefield (Huang and MacKerell Jr, 2013). The system was solvated using TIP3P (Jorgensen et al., 1983) water molecules in a cubic box such that the distance between any atom of the protein complex and the box edge was at least 10 Å. Subsequently, the system was neutralized (net charge = 0), and the salt concentration was brought to 0.15 M by adding 182 Na^+^ and 174 Cl^−^ ions. The simulations were performed under periodic boundary conditions and with the Particle Mesh Ewald (PME) method for calculating the long-range electrostatic interactions (Darden et al., 1993). The van der Waals interactions were smoothly switched off at 12 A°. Further, the solvated system was minimized (1,000 steps) to remove any steric clashes in the system. Following the minimization step, equilibration and production runs were performed with an integration time step of 2 fs, and all the bond lengths involving hydrogen atoms were fixed using the SHAKE algorithm (Andersen, 1983). The system was equilibrated using an NPT ensemble at 1 atm pressure and 310 K temperature with constraints, the production simulations were carried out for 500 ns without any constraints, and the trajectory was saved for every 10 picoseconds.

## Data availability statement

The original contributions presented in the study are included in the article/Supplementary material, further inquiries can be directed to the corresponding author.

## Author contributions

SP, IM, CM, and LM performed the experiments. SP, IM, CM, LM, TM, SN, and SB contributed to the experimental design, data analysis and interpretation, preparation of figures and tables, and preparation of the manuscript. All authors contributed to the article and approved the submitted version.

## Funding

This research was supported by grants from the National Institutes of Health R01AI083387 (SB) and R01GM137022 (SN).

## Conflict of interest

The authors declare that the research was conducted in the absence of any commercial or financial relationships that could be construed as a potential conflict of interest.

## Publisher’s note

All claims expressed in this article are solely those of the authors and do not necessarily represent those of their affiliated organizations, or those of the publisher, the editors and the reviewers. Any product that may be evaluated in this article, or claim that may be made by its manufacturer, is not guaranteed or endorsed by the publisher.

## References

[ref1] Abraham M. J. Murtola T. Schulz R. Páll S. Smith J. C. Hess B.. (2015). GROMACS: high performance molecular simulations through multi-level parallelism from laptops to supercomputers. SoftwareX 1-2, 19–25. doi: 10.1016/j.softx.2015.06.001

[ref2] Ahn V. E. Chu M. L.-H. Choi H.-J. Tran D. Abo A. Weis W. I. (2011). Structural basis of Wnt signaling inhibition by Dickkopf binding to LRP5/6. Dev. Cell 21, 862–873. doi: 10.1016/j.devcel.2011.09.003, PMID: 22000856 PMC3215855

[ref3] Andersen H. C. (1983). Rattle: A “velocity” version of the shake algorithm for molecular dynamics calculations. J. Comput. Phys. 52, 24–34. doi: 10.1016/0021-9991(83)90014-1

[ref4] Baggiolini M. Walz A. Kunkel S. L. (1989). Neutrophil-activating peptide-1/interleukin 8, a novel cytokine that activates neutrophils. J. Clin. Invest. 84, 1045–1049. doi: 10.1172/JCI114265, PMID: 2677047 PMC329758

[ref5] Basu M. Kota S. Banerjee A. K. Bose S. (2010). Role of human beta defensin 3 during type I interferon mediated antiviral response against vesicular stomatitis virus. Int. J. Interf. Cytokine Mediat. Res. 2, 23–32. doi: 10.2147/IJICMR.S6799

[ref6] Bazzoni F. Cassatella M. A. Rossi F. Ceska M. Dewald B. Baggiolini M. (1991). Phagocytosing neutrophils produce and release high amounts of the neutrophil-activating peptide 1/interleukin 8. J. Exp. Med. 173, 771–774. doi: 10.1084/jem.173.3.771, PMID: 1997655 PMC2118810

[ref7] Bedient L. Pokharel S. M. Chiok K. R. Mohanty I. Beach S. S. Miura T. A.. (2020). Lytic cell death mechanisms in human respiratory syncytial virus-infected macrophages: roles of pyroptosis and necroptosis. Viruses 12:932. doi: 10.3390/v12090932, PMID: 32854254 PMC7552060

[ref8] Biswas S. Friedland J. S. Remick D. G. Davies E. G. Sharland M. (1995). Elevated plasma interleukin 8 in respiratory syncytial virus bronchiolitis. Pediatr. Infect. Dis. J. 14:919. doi: 10.1097/00006454-199510000-00027, PMID: 8584330

[ref9] Bont L. Heijnen C. J. Kavelaars A. van Aalderen W. M. C. Brus F. Draaisma J. T.. (1999). Peripheral blood cytokine responses and disease severity in respiratory syncytial virus bronchiolitis. Eur. Respir. J. 14, 144–149. doi: 10.1034/j.1399-3003.1999.14a24.x, PMID: 10489842

[ref10] Bose S. Banerjee A. (2003). β-Catenin associates with human parainfluenza virus type 3 ribonucleoprotein complex and activates transcription of viral genome RNA in vitro. Gene Expr. J. Liver Res. 11, 241–249. doi: 10.3727/000000003783992252, PMID: 15200236 PMC5991151

[ref11] Bourhis E. Wang W. Tam C. Hwang J. Zhang Y. Spittler D.. (2011). Wnt antagonists bind through a short peptide to the first β-propeller domain of LRP5/6. Structure 19, 1433–1442. doi: 10.1016/j.str.2011.07.005, PMID: 21944579

[ref12] Bowie J. U. Lüthy R. Eisenberg D. (1991). A method to identify protein sequences that fold into a known three-dimensional structure. Science 253, 164–170. doi: 10.1126/science.1853201 1853201

[ref13] Ccgi M. (2016). Molecular operating environment (MOE), 2013.08. Chem. Comput. Gr. Inc., Montr. 354.

[ref14] Chang T.-H. Segovia J. Sabbah A. Mgbemena V. Bose S. (2012). Cholesterol-rich lipid rafts are required for release of infectious human respiratory syncytial virus particles. Virology 422, 205–213. doi: 10.1016/j.virol.2011.10.029, PMID: 22088217 PMC3249476

[ref15] Cheng Z. Biechele T. Wei Z. Morrone S. Moon R. T. Wang L.. (2011). Crystal structures of the extracellular domain of LRP6 and its complex with DKK1. Nat. Struct. Mol. Biol. 18, 1204–1210. doi: 10.1038/nsmb.2139, PMID: 21984209 PMC3249237

[ref16] Clément-Lacroix P. Ai M. Morvan F. Roman-Roman S. Vayssière B. Belleville C.. (2005). Lrp5-independent activation of Wnt signaling by lithium chloride increases bone formation and bone mass in mice. Proc. Natl. Acad. Sci. 102, 17406–17411. doi: 10.1073/pnas.0505259102, PMID: 16293698 PMC1297659

[ref17] Clevers H. (2006). Wnt/β-catenin signaling in development and disease. Cells 127, 469–480. doi: 10.1016/j.cell.2006.10.018 17081971

[ref18] Colavita I. Nigro E. Sarnataro D. Scudiero O. Granata V. Daniele A.. (2015). Membrane protein 4F2/CD98 is a cell surface receptor involved in the internalization and trafficking of human β-Defensin 3 in epithelial cells. Chem. Biol. 22, 217–228. doi: 10.1016/j.chembiol.2014.11.020, PMID: 25641165

[ref19] Colovos C. Yeates T. O. (1993). Verification of protein structures: patterns of nonbonded atomic interactions. Protein Sci. 2, 1511–1519. doi: 10.1002/pro.5560020916, PMID: 8401235 PMC2142462

[ref20] Corsello T. Qu Y. Ivanciuc T. Garofalo R. P. Casola A. (2022). Antiviral activity of extracellular vesicles derived from respiratory syncytial virus-infected airway epithelial cells. Front. Immunol. 13:13. doi: 10.3389/fimmu.2022.886701 PMC9412241 36032066

[ref21] Darden T. York D. Pedersen L. (1993). Particle mesh Ewald: an N· log (N) method for Ewald sums in large systems. J. Chem. Phys. 98, 10089–10092. doi: 10.1063/1.464397

[ref22] Dijksterhuis J. P. Baljinnyam B. Stanger K. Sercan H. O. Ji Y. Andres O.. (2015). Systematic mapping of WNT-FZD protein interactions reveals functional selectivity by distinct WNT-FZD pairs. J. Biol. Chem. 290, 6789–6798. doi: 10.1074/jbc.M114.612648, PMID: 25605717 PMC4358105

[ref23] Diseases C. (1993). Use of ribavirin in the treatment of respiratory syncytial virus infection. Pediatrics 92, 501–504. doi: 10.1542/peds.92.3.501 8361820

[ref24] Emboriadou M. Hatzistilianou M. Magnisali C. Sakelaropoulou A. Exintari M. Conti P.. (2007). Human neutrophil elastase in RSV bronchiolitis. Ann. Clin. Lab. Sci. 37, 79–84. PMID: 17311874

[ref25] Everard M. L. Swarbrick A. Wrightham M. McIntyre J. Dunkley C. James P. D.. (1994). Analysis of cells obtained by bronchial lavage of infants with respiratory syncytial virus infection. Arch. Dis. Child. 71, 428–432. doi: 10.1136/adc.71.5.428, PMID: 7826113 PMC1030058

[ref26] Falsey A. R. Hennessey P. A. Formica M. A. Cox C. Walsh E. E. (2005). Respiratory syncytial virus infection in elderly and high-risk adults. N. Engl. J. Med. 352, 1749–1759. doi: 10.1056/Nejmoa043951 15858184

[ref27] Fiedler M. A. Wernke-Dollries K. Stark J. M. (1995). Respiratory syncytial virus increases IL-8 gene expression and protein release in A549 cells. Am. J. Physiol. Cell. Mol. Physiol. 269, L865–L872. doi: 10.1152/ajplung.1995.269.6.L865 8572249

[ref28] Foronjy R. F. Ochieng P. O. Salathe M. A. Dabo A. J. Eden E. Baumlin N.. (2016). Protein tyrosine phosphatase 1B negatively regulates S100A9-mediated lung damage during respiratory syncytial virus exacerbations. Mucosal Immunol. 9, 1317–1329. doi: 10.1038/mi.2015.138, PMID: 26813343 PMC4963308

[ref29] Fruitwala S. El-Naccache D. W. Chang T. L. (2019). Multifaceted immune functions of human defensins and underlying mechanisms. Seminars Cell Dev. Biol. 88, 163–172. doi: 10.1016/j.semcdb.2018.02.023, PMID: 29501617 PMC6485945

[ref30] Gonsalves F. C. Klein K. Carson B. B. Katz S. Ekas L. A. Evans S.. (2011). An RNAi-based chemical genetic screen identifies three small-molecule inhibitors of the Wnt/wingless signaling pathway. Proc. Natl. Acad. Sci. 108, 5954–5963. doi: 10.1073/pnas.1017496108, PMID: 21393571 PMC3076864

[ref31] Graham B. S. Anderson L. J. (2013). Challenges and opportunities for respiratory syncytial virus vaccines. Challenges Oppor. Respir. Syncytial Virus Vaccines 372, 391–404. doi: 10.1007/978-3-642-38919-1_20, PMID: 24362701 PMC7121045

[ref32] Griffiths C. Drews S. J. Marchant D. J. (2017). Respiratory syncytial virus: infection, detection, and new options for prevention and treatment. Clin. Microbiol. Rev. 30, 277–319. doi: 10.1128/CMR.00010-16 27903593 PMC5217795

[ref33] Hillary V. E. Ceasar S. A. (2022). A review on the mechanism and applications of CRISPR/Cas9/Cas12/Cas13/Cas14 proteins utilized for genome engineering. Mol. Biotechnol. 65, 1–15. doi: 10.1007/s12033-022-00567-0 36163606 PMC9512960

[ref34] Hillyer P. Shepard R. Uehling M. Krenz M. Sheikh F. Thayer K. R.. (2018). Differential responses by human respiratory epithelial cell lines to respiratory syncytial virus reflect distinct patterns of infection control. J. Virol. 92, e02202–e02217. doi: 10.1128/JVI.02202-17 29769339 PMC6052282

[ref35] Hirai H. Matoba K. Mihara E. Arimori T. Takagi J. (2019). Crystal structure of a mammalian Wnt–frizzled complex. Nat. Struct. Mol. Biol. 26, 372–379. doi: 10.1038/s41594-019-0216-z, PMID: 31036956

[ref36] Honorato R. V. Koukos P. I. Jiménez-García B. Tsaregorodtsev A. Verlato M. Giachetti A.. (2021). Structural biology in the clouds: the WeNMR-EOSC ecosystem. Front. Mol. Biosci. 8:729513. doi: 10.3389/fmolb.2021.729513, PMID: 34395534 PMC8356364

[ref37] Hosakote Y. M. Liu T. Castro S. M. Garofalo R. P. Casola A. (2009). Respiratory syncytial virus induces oxidative stress by modulating antioxidant enzymes. Am. J. Respir. Cell Mol. Biol. 41, 348–357. doi: 10.1165/rcmb.2008-0330OC, PMID: 19151318 PMC2742754

[ref38] Hotard A. L. Shaikh F. Y. Lee S. Yan D. Teng M. N. Plemper R. K.. (2012). A stabilized respiratory syncytial virus reverse genetics system amenable to recombination-mediated mutagenesis. Virology 434, 129–136. doi: 10.1016/j.virol.2012.09.022 23062737 PMC3492879

[ref39] Hsu P. D. Lander E. S. Zhang F. (2014). Development and applications of CRISPR-Cas9 for genome engineering. Cells 157, 1262–1278. doi: 10.1016/j.cell.2014.05.010, PMID: 24906146 PMC4343198

[ref40] Huang J. MacKerell A. D. Jr. (2013). CHARMM36 all-atom additive protein force field: validation based on comparison to NMR data. J. Comput. Chem. 34, 2135–2145. doi: 10.1002/jcc.23354, PMID: 23832629 PMC3800559

[ref41] Huang L. Xiang M. Ye P. Zhou W. Chen M. (2018). Beta-catenin promotes macrophage-mediated acute inflammatory response after myocardial infarction. Immunol. Cell Biol. 96, 100–113. doi: 10.1111/imcb.1019, PMID: 29356094

[ref42] Hurwitz J. L. (2011). Respiratory syncytial virus vaccine development. Expert Rev. Vaccines 10, 1415–1433. doi: 10.1586/erv.11.120, PMID: 21988307 PMC3255794

[ref43] Imai Y. Kuba K. Neely G. G. Yaghubian-Malhami R. Perkmann T. van Loo G.. (2008). Identification of oxidative stress and toll-like receptor 4 signaling as a key pathway of acute lung injury. Cells 133, 235–249. doi: 10.1016/j.cell.2008.02.043, PMID: 18423196 PMC7112336

[ref44] Jamaluddin M. Garofalo R. Ogra P. L. Brasier A. R. (1996). Inducible translational regulation of the NF-IL6 transcription factor by respiratory syncytial virus infection in pulmonary epithelial cells. J. Virol. 70, 1554–1563. doi: 10.1128/jvi.70.3.1554-1563.1996, PMID: 8627674 PMC189977

[ref45] Janda C. Y. Dang L. T. You C. Chang J. de Lau W. Zhong Z. A.. (2017). Surrogate Wnt agonists that phenocopy canonical Wnt and β-catenin signalling. Nature 545, 234–237. doi: 10.1038/nature22306, PMID: 28467818 PMC5815871

[ref46] Janda C. Y. Waghray D. Levin A. M. Thomas C. Garcia K. C. (2012). Structural basis of Wnt recognition by frizzled. Science 337, 59–64. doi: 10.1126/science.1222879 22653731 PMC3577348

[ref47] Jang J. Ha J.-H. Chung S.-I. Yoon Y. (2014). β-Catenin regulates NF-κB activity and inflammatory cytokine expression in bronchial epithelial cells treated with lipopolysaccharide. Int. J. Mol. Med. 34, 632–638. doi: 10.3892/ijmm.2014.1807, PMID: 24938929

[ref48] Jorgensen W. L. Chandrasekhar J. Madura J. D. Impey R. W. Klein M. L. (1983). Comparison of simple potential functions for simulating liquid water. J. Chem. Phys. 79, 926–935. doi: 10.1063/1.445869

[ref49] Kong X. San Juan H. Behera A. Peeples M. E. Wu J. Lockey R. F.. (2004). ERK-1/2 activity is required for efficient RSV infection. FEBS Lett. 559, 33–38. doi: 10.1016/S0014-5793(04)00002-X, PMID: 14960303

[ref50] Kota S. Sabbah A. Harnack R. Xiang Y. Meng X. Bose S. (2008). Role of human β-defensin-2 during tumor necrosis factor-α/NF-κB-mediated innate antiviral response against human respiratory syncytial virus. J. Biol. Chem. 283, 22417–22429. doi: 10.1074/jbc.M710415200, PMID: 18567888 PMC2504899

[ref51] Lee J. Cheng X. Swails J. M. Yeom M. S. Eastman P. K. Lemkul J. A.. (2016). CHARMM-GUI input generator for NAMD, GROMACS, AMBER, OpenMM, and CHARMM/OpenMM simulations using the CHARMM36 additive force field. J. Chem. Theory Comput. 12, 405–413. doi: 10.1021/acs.jctc.5b00935, PMID: 26631602 PMC4712441

[ref52] Lee M. G. Oh H. Park J. W. You J. S. Han J.-W. (2022). Nuclear S6K1 enhances oncogenic Wnt signaling by inducing Wnt/β-catenin transcriptional complex formation. Int. J. Mol. Sci. 23:16143. doi: 10.3390/ijms232416143, PMID: 36555784 PMC9785994

[ref53] Lévy L. Neuveut C. Renard C.-A. Charneau P. Branchereau S. Gauthier F.. (2002). Transcriptional activation of interleukin-8 by β-catenin-Tcf4. J. Biol. Chem. 277, 42386–42393. doi: 10.1074/jbc.M207418200, PMID: 12200448

[ref54] Lin Y. Ohkawara B. Ito M. Misawa N. Miyamoto K. Takegami Y.. (2016). Molecular hydrogen suppresses activated Wnt/β-catenin signaling. Sci. Rep. 6, 1–14. doi: 10.1038/srep31986 27558955 PMC5001535

[ref55] Liu J. Xiao Q. Xiao J. Niu C. Li Y. Zhang X.. (2022). Wnt/β-catenin signalling: function, biological mechanisms, and therapeutic opportunities. Signal Transduct. Target. Ther. 7:3. doi: 10.1038/s41392-021-00762-6, PMID: 34980884 PMC8724284

[ref56] Lüthy R. Bowie J. U. Eisenberg D. (1992). Assessment of protein models with three-dimensional profiles. Nature 356, 83–85. doi: 10.1038/356083a0 1538787

[ref57] Ma B. Hottiger M. O. (2016). Crosstalk between Wnt/β-catenin and NF-κB signaling pathway during inflammation. Front. Immunol. 7:378. doi: 10.3389/fimmu.2016.00378, PMID: 27713747 PMC5031610

[ref58] Masckauchan T. N. (2005). Shawber CJ, Funahashi Y, Li CM, Kitajewski J. Wnt/beta-catenin signal. induces proliferation, Surviv. Interleukin-8 hum. Endothel. Cells Angiogenes. 8, 43–51. doi: 10.1007/s10456-005-5612-9 16132617

[ref59] Mastronarde J. G. He R. Monick M. M. Mukaida N. Matsushima K. Hunninghake G. W. (1996). Induction of interleukin (IL)-8 gene expression by respiratory syncytial virus involves activation of nuclear factor (NF)-κB and NF-IL-6. J. Infect. Dis. 174, 262–267. doi: 10.1093/infdis/174.2.262, PMID: 8699053

[ref60] Matoba K. Mihara E. Tamura-Kawakami K. Miyazaki N. Maeda S. Hirai H.. (2017). Conformational freedom of the LRP6 ectodomain is regulated by N-glycosylation and the binding of the Wnt antagonist Dkk1. Cell Rep. 18, 32–40. doi: 10.1016/j.celrep.2016.12.017, PMID: 28052259

[ref61] McNamara P. S. Ritson P. Selby A. Hart C. A. Smyth R. L. (2003). Bronchoalveolar lavage cellularity in infants with severe respiratory syncytial virus bronchiolitis. Arch. Dis. Child. 88, 922–926. doi: 10.1136/adc.88.10.922, PMID: 14500316 PMC1719332

[ref62] Meng J. Lee S. Hotard A. L. Moore M. L. (2014). Refining the balance of attenuation and immunogenicity of respiratory syncytial virus by targeted codon deoptimization of virulence genes. MBio 5, e01704–e01714. doi: 10.1128/mBio.01704-14, PMID: 25249281 PMC4173764

[ref63] Mgbemena V. Segovia J. Chang T.-H. Bose S. (2013). KLF6 and iNOS regulates apoptosis during respiratory syncytial virus infection. Cell. Immunol. 283, 1–7. doi: 10.1016/j.cellimm.2013.06.002, PMID: 23831683 PMC3744625

[ref64] Mgbemena V. Segovia J. A. Chang T.-H. Tsai S.-Y. Cole G. T. Hung C.-Y.. (2012). Transactivation of inducible nitric oxide synthase gene by Kruppel-like factor 6 regulates apoptosis during influenza A virus infection. J. Immunol. 189, 606–615. doi: 10.4049/jimmunol.1102742, PMID: 22711891 PMC3392426

[ref65] More S. Yang X. Zhu Z. Bamunuarachchi G. Guo Y. Huang C.. (2018). Regulation of influenza virus replication by Wnt/β-catenin signaling. PLoS One 13:e0191010. doi: 10.1371/journal.pone.0191010, PMID: 29324866 PMC5764324

[ref66] Murawski M. R. Bowen G. N. Cerny A. M. Anderson L. J. Haynes L. M. Tripp R. A.. (2009). Respiratory syncytial virus activates innate immunity through toll-like receptor 2. J. Virol. 83, 1492–1500. doi: 10.1128/JVI.00671-08, PMID: 19019963 PMC2620898

[ref67] Musunuru K. (2017). The hope and hype of CRISPR-Cas9 genome editing: a review. JAMA Cardiol. 2, 914–919. doi: 10.1001/jamacardio.2017.1713, PMID: 28614576

[ref68] Nair H. Nokes D. J. Gessner B. D. Dherani M. Madhi S. A. Singleton R. J.. (2010). Global burden of acute lower respiratory infections due to respiratory syncytial virus in young children: a systematic review and meta-analysis. Lancet 375, 1545–1555. doi: 10.1016/S0140-6736(10)60206-1, PMID: 20399493 PMC2864404

[ref69] Pontius J. Richelle J. Wodak S. J. (1996). Deviations from standard atomic volumes as a quality measure for protein crystal structures. J. Mol. Biol. 264, 121–136. doi: 10.1006/jmbi.1996.0628, PMID: 8950272

[ref70] Rajan A. Piedra F.-A. Aideyan L. McBride T. Robertson M. Johnson H. L.. (2022). Multiple respiratory syncytial virus (RSV) strains infecting HEp-2 and A549 cells reveal cell line-dependent differences in resistance to RSV infection. J. Virol. 96, e01904–e01921. doi: 10.1128/jvi.01904-21 35285685 PMC9006923

[ref71] Ran F. A. Hsu P. D. Wright J. Agarwala V. Scott D. A. Zhang F. (2013). Genome engineering using the CRISPR-Cas9 system. Nat. Protoc. 8, 2281–2308. doi: 10.1038/nprot.2013.143, PMID: 24157548 PMC3969860

[ref72] Ren Q. Chen J. Liu Y. (2021). LRP5 and LRP6 in Wnt signaling: similarity and divergence. Front. Cell Dev. Biol. 9:670960. doi: 10.3389/fcell.2021.670960, PMID: 34026761 PMC8134664

[ref73] Rim E. Y. Clevers H. Nusse R. (2022). The Wnt pathway: from signaling mechanisms to synthetic modulators. Annu. Rev. Biochem. 91, 571–598. doi: 10.1146/annurev-biochem-040320-103615, PMID: 35303793

[ref74] Romero J. R. (2003). Palivizumab prophylaxis of respiratory syncytial virus disease from 1998 to 2002: results from four years of palivizumab usage. Pediatr. Infect. Dis. J. 22, S46–S54. doi: 10.1097/01.inf.0000053885.34703.84, PMID: 12671452

[ref75] Romero C. A. Remor A. Latini A. De Paul A. L. Torres A. I. Mukdsi J. H. (2017). Uric acid activates NRLP3 inflammasome in an in-vivo model of epithelial to mesenchymal transition in the kidney. J. Mol. Histol. 48, 209–218. doi: 10.1007/s10735-017-9720-9, PMID: 28374152

[ref76] Rudd B. D. Burstein E. Duckett C. S. Li X. Lukacs N. W. (2005). Differential role for TLR3 in respiratory syncytial virus-induced chemokine expression. J. Virol. 79, 3350–3357. doi: 10.1128/JVI.79.6.3350-3357.2005, PMID: 15731229 PMC1075725

[ref77] Russell C. D. Unger S. A. Walton M. Schwarze J. (2017). The human immune response to respiratory syncytial virus infection. Clin. Microbiol. Rev. 30, 481–502. doi: 10.1128/CMR.00090-16, PMID: 28179378 PMC5355638

[ref78] Ruuskanen O. Lahti E. Jennings L. C. Murdoch D. R. (2011). Viral pneumonia. Lancet 377, 1264–1275. doi: 10.1016/S0140-6736(10)61459-6, PMID: 21435708 PMC7138033

[ref79] Sebina I. Phipps S. (2020). The contribution of neutrophils to the pathogenesis of RSV bronchiolitis. Viruses 12:808. doi: 10.3390/v12080808, PMID: 32726921 PMC7472258

[ref80] Semple F. Webb S. Li H. Patel H. B. Perretti M. Jackson I. J.. (2010). Human β-defensin 3 has immunosuppressive activity in vitro and in vivo. Eur. J. Immunol. 40, 1073–1078. doi: 10.1002/eji.200940041, PMID: 20104491 PMC2948537

[ref81] Sharma A. Yang W.-L. Ochani M. Wang P. (2017). Mitigation of sepsis-induced inflammatory responses and organ injury through targeting Wnt/β-catenin signaling. Sci. Rep. 7:9235. doi: 10.1038/s41598-017-08711-6, PMID: 28835626 PMC5569053

[ref82] Shelley J. R. Davidson D. J. Dorin J. R. (2020). The dichotomous responses driven by β-defensins. Front. Immunol. 11:1176. doi: 10.3389/fimmu.2020.01176, PMID: 32595643 PMC7304343

[ref83] Shen M. Sali A. (2006). Statistical potential for assessment and prediction of protein structures. Protein Sci. 15, 2507–2524. doi: 10.1110/ps.062416606, PMID: 17075131 PMC2242414

[ref84] Shirato K. Ujike M. Kawase M. Matsuyama S. (2012). Increased replication of respiratory syncytial virus in the presence of cytokeratin 8 and 18. J. Med. Virol. 84, 365–370. doi: 10.1002/jmv.23196, PMID: 22170560 PMC7166714

[ref85] Su F. Chen X. Liu X. Liu G. Zhang Y. (2018). Expression of recombinant HBD3 protein that reduces mycobacterial infection capacity. AMB Express 8, 1–9. doi: 10.1186/s13568-018-0573-8 29556853 PMC5861256

[ref86] Thomas L. H. Wickremasinghe M. I. Y. Sharland M. Friedland J. S. (2000). Synergistic upregulation of interleukin-8 secretion from pulmonary epithelial cells by direct and monocyte-dependent effects of respiratory syncytial virus infection. J. Virol. 74, 8425–8433. doi: 10.1128/JVI.74.18.8425-8433.2000, PMID: 10954542 PMC116353

[ref87] Trujano-Camacho S. Cantú-de León D. Delgado-Waldo I. Coronel-Hernández J. Millan-Catalan O. Hernández-Sotelo D.. (2021). Inhibition of Wnt-β-catenin signaling by ICRT14 drug depends of post-transcriptional regulation by HOTAIR in human cervical cancer HeLa cells. Front. Oncol. 11:729228. doi: 10.3389/fonc.2021.729228 34778043 PMC8580948

[ref88] Tsutsumi N. Mukherjee S. Waghray D. Janda C. Y. Jude K. M. Miao Y.. (2020). Structure of human Frizzled5 by fiducial-assisted cryo-EM supports a heterodimeric mechanism of canonical Wnt signaling. elife 9:e58464. doi: 10.7554/eLife.58464, PMID: 32762848 PMC7442489

[ref89] Varadi M. Anyango S. Deshpande M. Nair S. Natassia C. Yordanova G.. (2022). AlphaFold protein structure database: massively expanding the structural coverage of protein-sequence space with high-accuracy models. Nucleic Acids Res. 50, D439–D444. doi: 10.1093/nar/gkab1061, PMID: 34791371 PMC8728224

[ref90] Wang S.-W. Gao C. Zheng Y.-M. Yi L. Lu J.-C. Huang X.-Y.. (2022). Current applications and future perspective of CRISPR/Cas9 gene editing in cancer. Mol. Cancer 21, 1–27. doi: 10.1186/s12943-022-01518-8 35189910 PMC8862238

[ref91] Webb B. Sali A. (2016). Comparative protein structure modeling using MODELLER. Curr. Protoc. Bioinformatics 54, 5–6. doi: 10.1002/cpbi.3 PMC5031415 27322406

[ref92] Wright S. C. Kozielewicz P. Kowalski-Jahn M. Petersen J. Bowin C.-F. Slodkowicz G.. (2019). A conserved molecular switch in class F receptors regulates receptor activation and pathway selection. Nat. Commun. 10:667. doi: 10.1038/s41467-019-08630-2, PMID: 30737406 PMC6368630

[ref93] Xu D. Lu W. (2020). Defensins: a double-edged sword in host immunity. Front. Immunol. 11:764. doi: 10.3389/fimmu.2020.00764, PMID: 32457744 PMC7224315

[ref94] Zebisch M. Jackson V. A. Zhao Y. Jones E. Y. (2016). Structure of the dual-mode Wnt regulator Kremen1 and insight into ternary complex formation with LRP6 and Dickkopf. Structure 24, 1599–1605. doi: 10.1016/j.str.2016.06.020, PMID: 27524201 PMC5014086

[ref95] Zhan T. Rindtorff N. Boutros M. (2017). Wnt signaling in cancer. Oncogene 36, 1461–1473. doi: 10.1038/onc.2016.304, PMID: 27617575 PMC5357762

[ref96] Zhao W. Sun Z. Wang S. Li Z. Zheng L. (2015). Wnt1 participates in inflammation induced by lipopolysaccharide through upregulating scavenger receptor A and NF-kB. Inflammation 38, 1700–1706. doi: 10.1007/s10753-015-0147-8, PMID: 25749569 PMC4495710

[ref97] Zhou J. Zhang Y. Li L. Fu H. Yang W. Yan F. (2018). Human β-defensin 3-combined gold nanoparticles for enhancement of osteogenic differentiation of human periodontal ligament cells in inflammatory microenvironments. Int. J. Nanomedicine 13, 555–567. doi: 10.2147/IJN.S150897, PMID: 29416335 PMC5790078

[ref98] Zhu Y. (2022). Advances in CRISPR/Cas9. Biomed. Res. Int. 2022, 1–13. doi: 10.1155/2022/9978571

