# Supplementary material for: Human beta defensin-3 mediated activation of β-catenin during human respiratory syncytial virus infection: interaction of HBD3 with LDL receptor-related protein 5
Source: Front Microbiol. 2023 Jun 22;14:1186510. doi: 10.3389/fmicb.2023.1186510 (PMC10324619; doi:10.3389/fmicb.2023.1186510)
Supplement: Supplementary file 1 [file Data_Sheet_1.pdf]

Supplementary Material

Human beta defensin-3 mediated activation of β-catenin during human respiratory syncytial virus infection: Interaction of HBD3 with LDL receptor-related protein 5

Swechha M. Pokharel[1*$^], Indira Mohanty[1*], Charles Mariasoosai[2], Tanya A. Miura[3], Lisette A. Maddison[4], Senthil Natesan[2¶], Santanu Bose[1¶#]

[#]Corresponding author: santanu.bose@wsu.edu

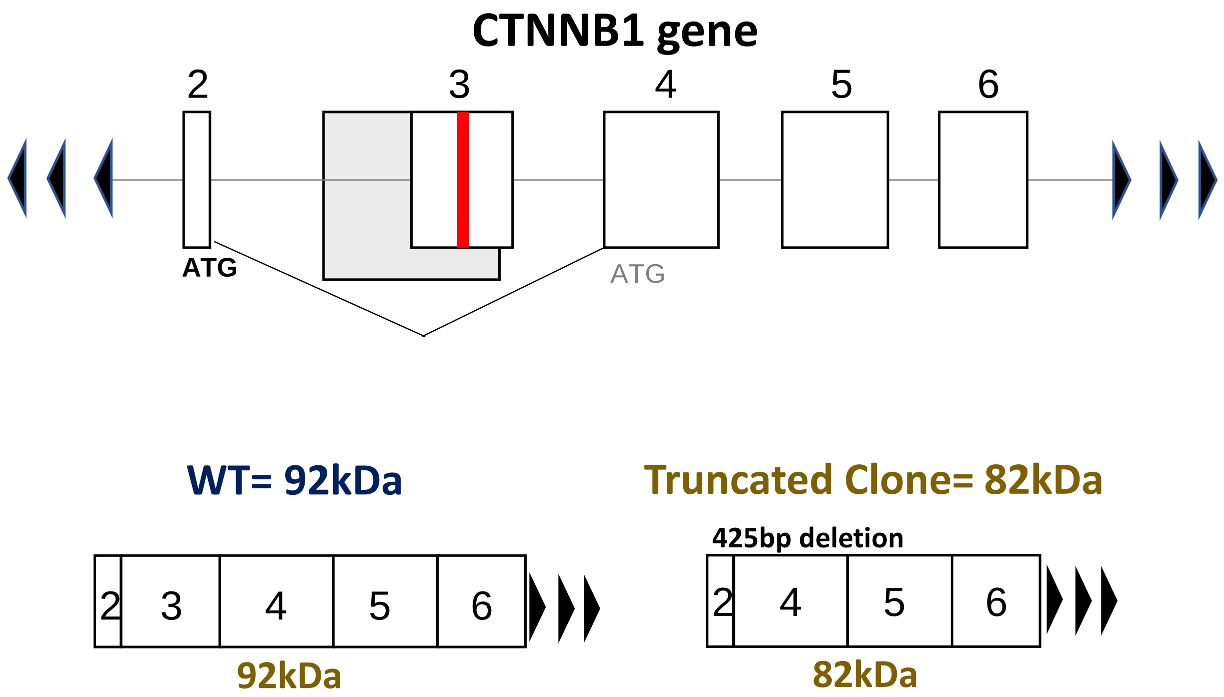

Supplementary Figure 1. Upper schematic depicts the partial genomic structure of β-catenin protein encoding CTNNB1 gene. The red line indicates the location of the Cas9 target. The grey box indicates the region removed with the large deletion in the truncated clone. Exon 3 would be skipped leading to a truncated protein.

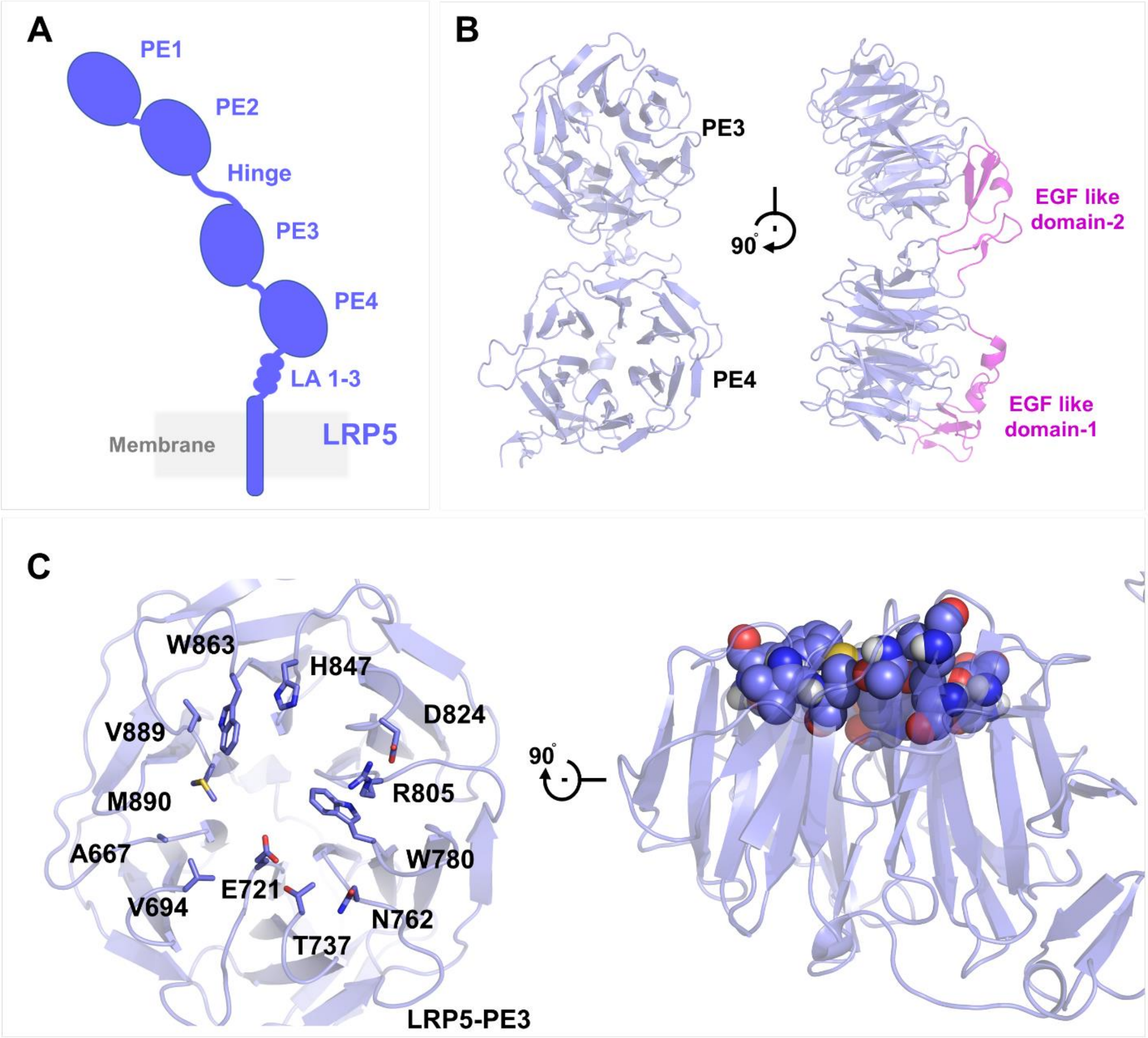

Supplementary Figure 2. Structure of LRP5. A) Schematic representation of the LRP5 structure. LPR5 consists of four β-propeller domains connected by EGF (Epidermal Growth Factor) like domains, three LA (LDLR type A) domains, and a transmembrane helix. B) Structure of third and fourth β-propeller domains. EGF-like domain interconnects the β-propeller domains. A pair of β-propeller and the EGF domain together is termed as PE domain. C-D) Binding site residues at the PE3 domain surface. The residues were shown as sticks (C) and in CPK representations (D).

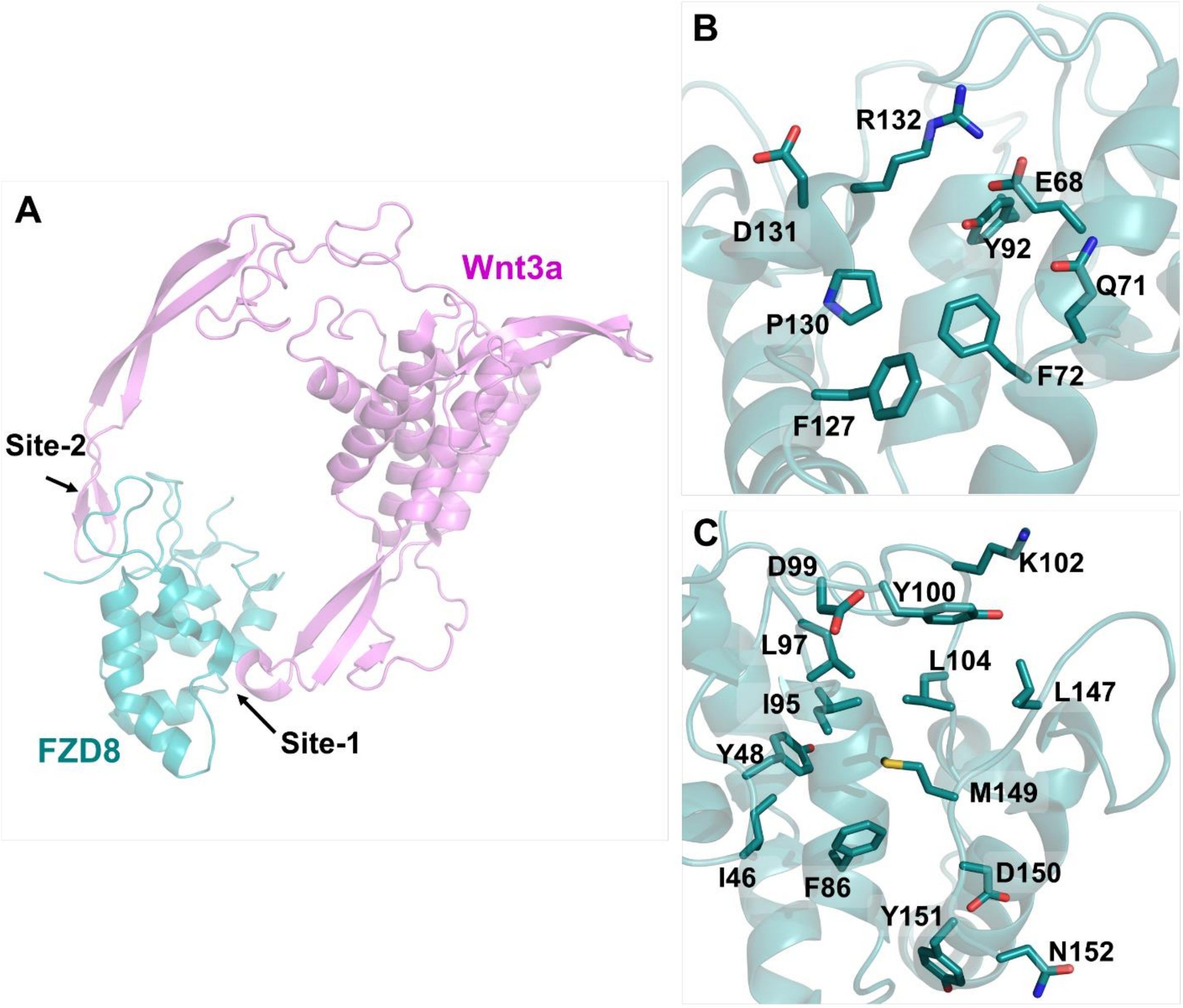

Supplementary Figure 3. Structure of FZD8 with Wnt3a. A) Wnt binds to the FZD8 receptor by making contacts at two different sites. The residues at site-1 and site-2 in contact with WNT3a are shown in stick representations in B and C, respectively.

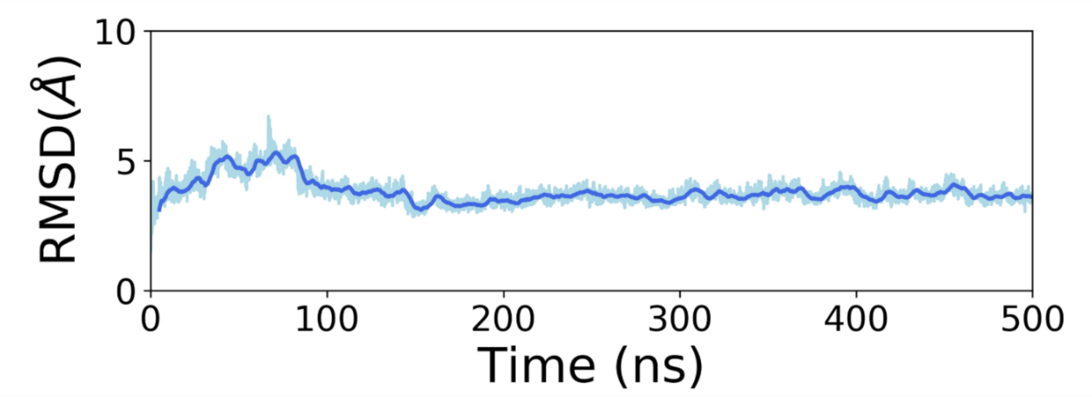

Supplementary Figure 4. RMSD plot of the LRP5-HBD3-FZD8 complex during the 500 ns MD simulation. The plot indicates that the complex becomes stable after 100 ns of the simulation.

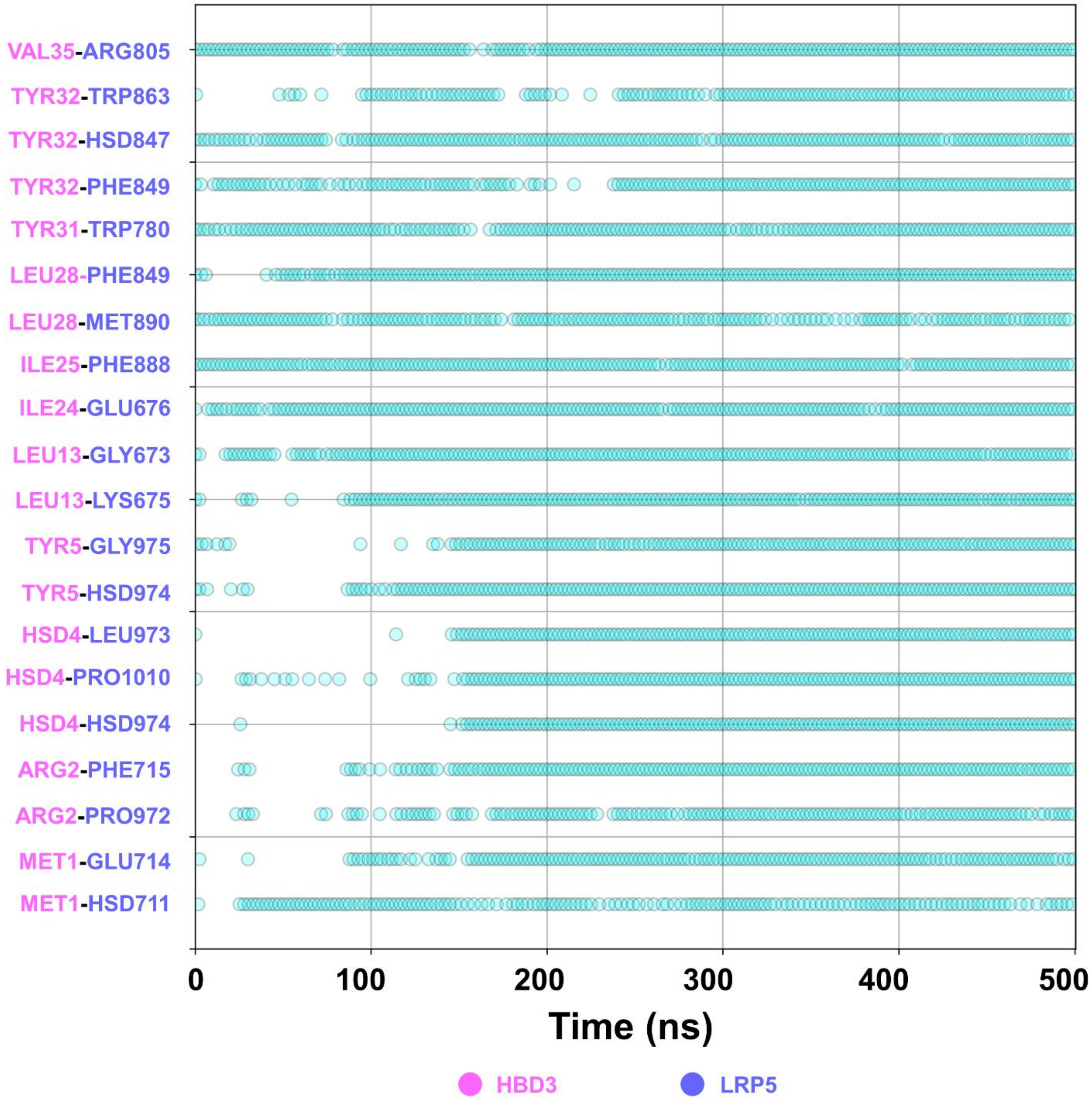

Supplementary Figure 5. Residue contacts at the interaction interface between LRP5 and HBD3. The residues from the interacting partners within 4 Å distance were monitored, and those with contact occupancy of 80% or more were displayed here. These contacts were intact for more than 80% of the simulation time, 500 ns. The residues from HBD3 and LRP5 were shown in magenta and purple-blue colors, respectively.

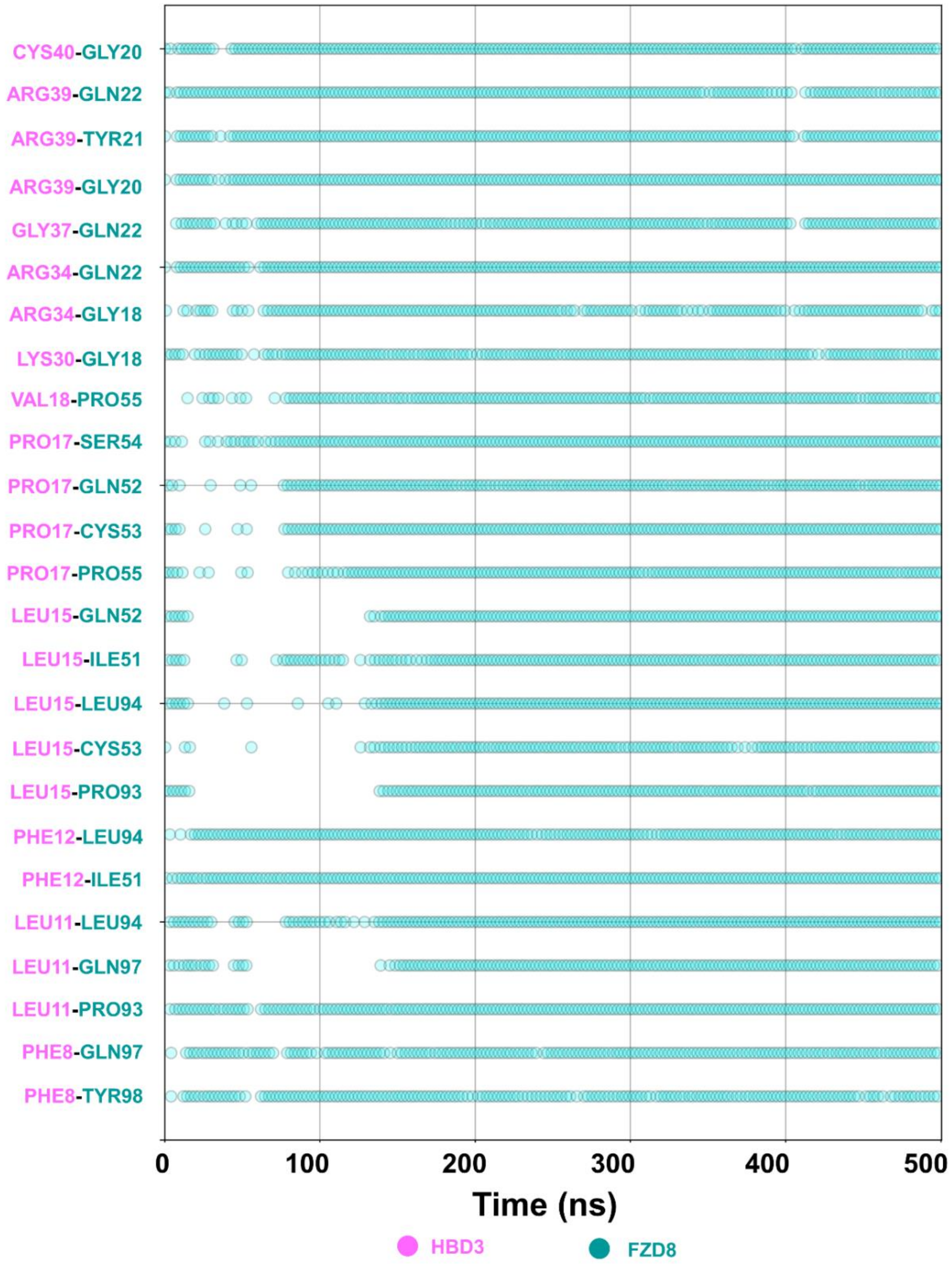

Supplementary Figure 6. Residue contacts at the interaction interface between HBD3 and FZD8. The residues from the interacting partners within 4 Å distance were monitored, and those with contact occupancy of 80% or more were displayed here. These contacts were intact for more than 80% of the simulation time, 500 ns. The residues from HBD3 and FZD8 were shown in magenta and teal colors, respectively.

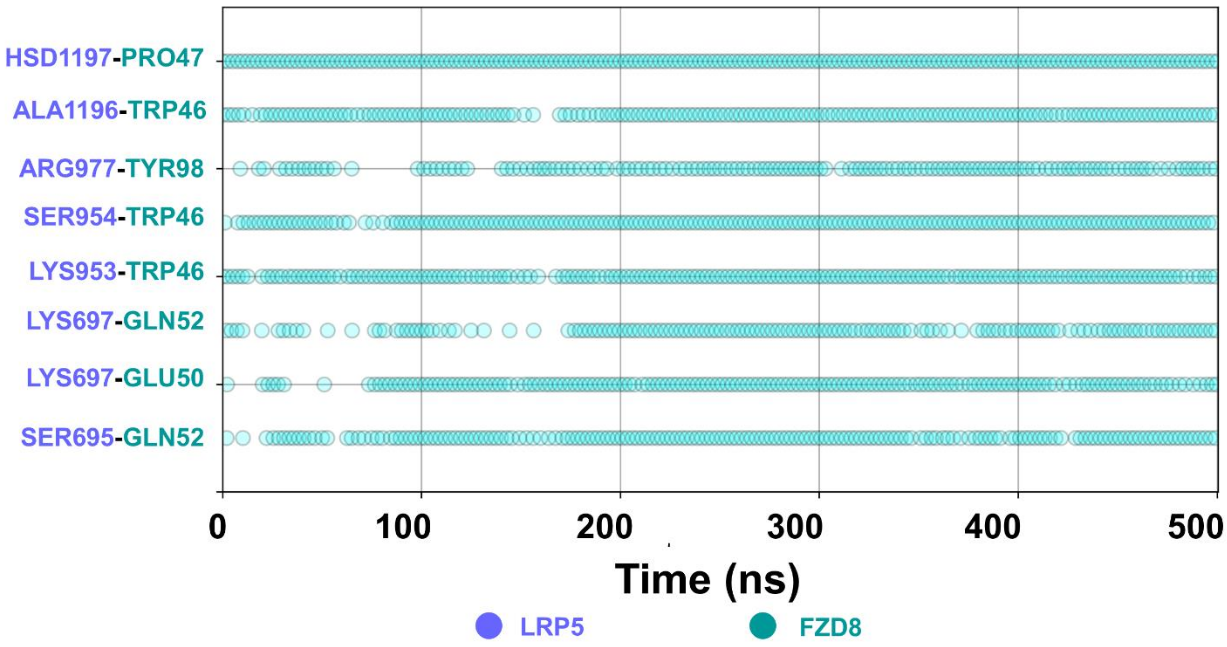

Supplementary Figure 7. Residue contacts at the interaction interface between LRP5 and FZD8. The residues from the interacting partners within 4 Å distance were monitored, and those with contact occupancy of 80% or more were displayed here. These contacts were intact for more than 80% of the simulation time, 500 ns. The residues from LRP5 and FZD8 were shown in magenta and purple and teal colors, respectively.

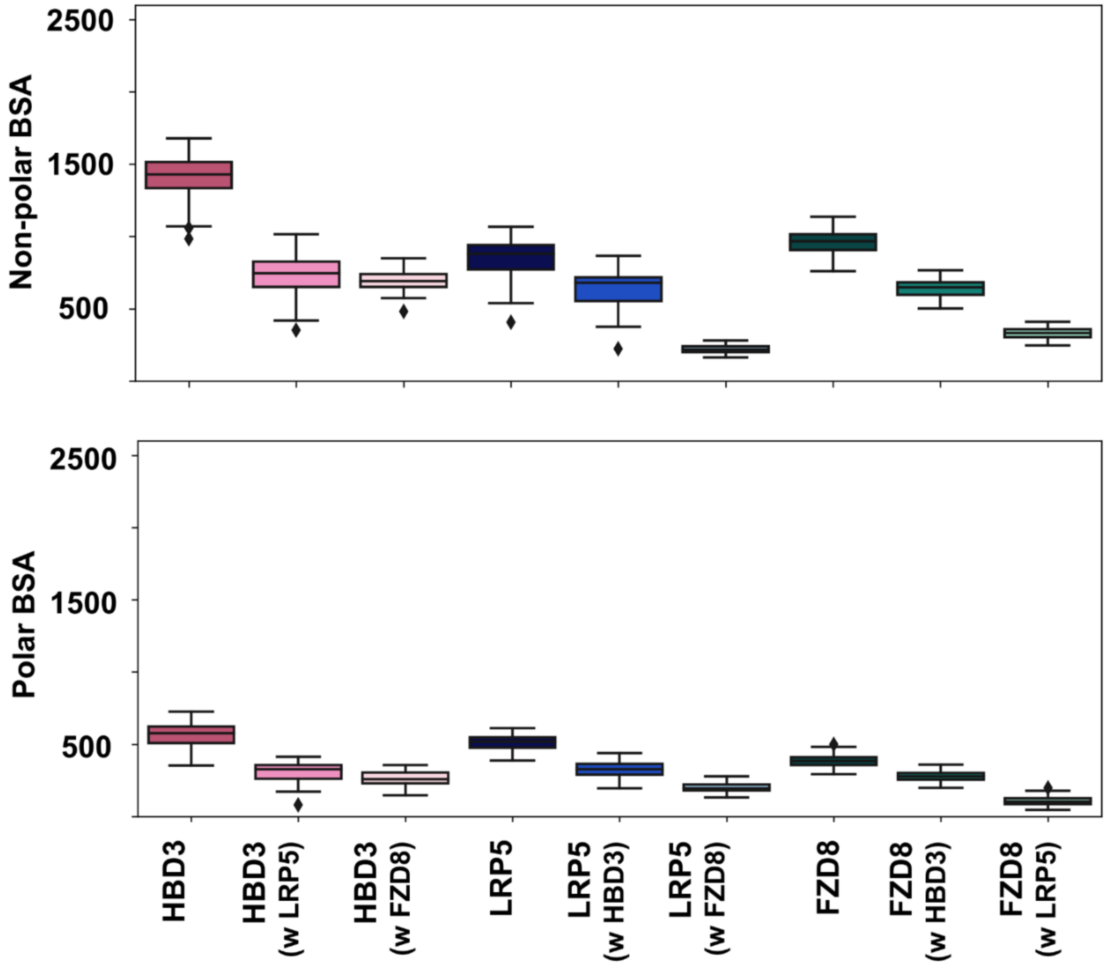

Supplementary Figure 8. The nonpolar and polar buried surface areas of LRP5, HBD3, and FZD8. The amount of solvent-accessible surface area of interacting partners lost during the binding process has been shown to correlate well with the binding affinity in the literature. The total solvent-accessible surface area (polar and nonpolar) and the fraction of buried surface area (BSA) are shown for each binding partner. The graphs indicate that significant fractions of nonpolar surface area were involved in the complex formation.

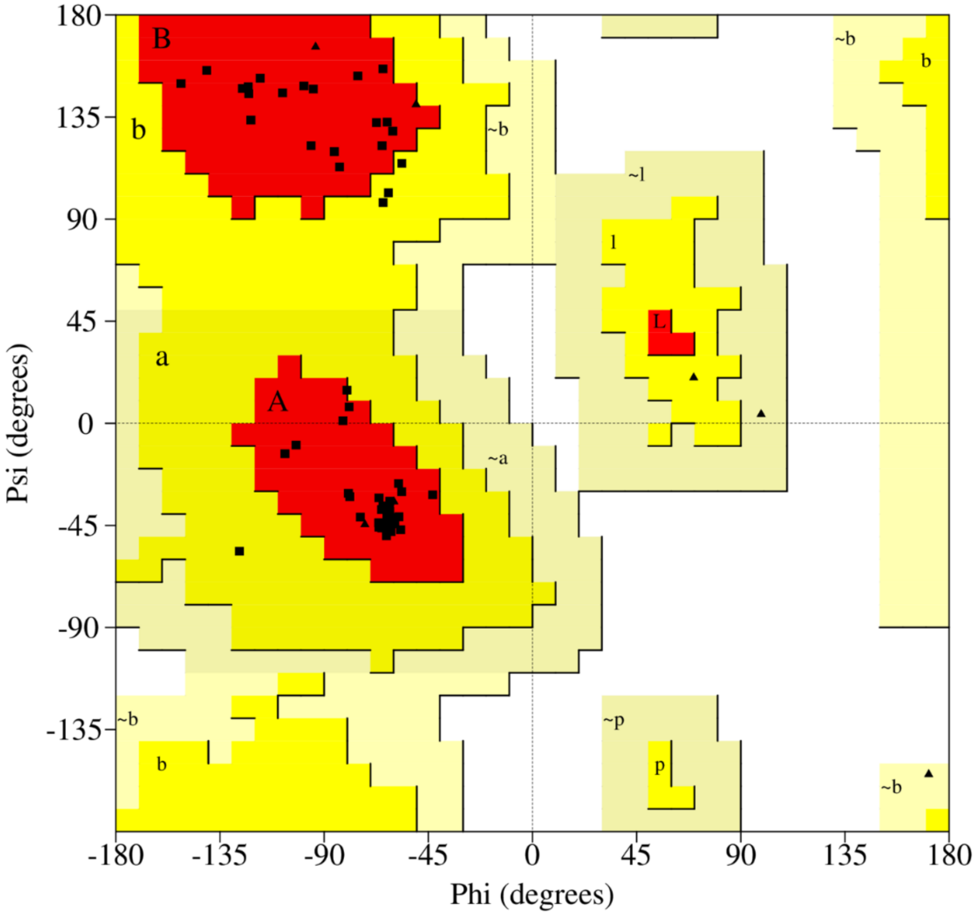

Supplementary Figure 9. Ramachandran plot for the homology structure of HBD3 from the AlphaFold model database. 96.4% of the residues are present in the most favored region, and 3.6 % of the residues are present in the additionally allowed region. Almost all the residues predicted to be in the allowed region represent the better stereochemical quality of the structure.

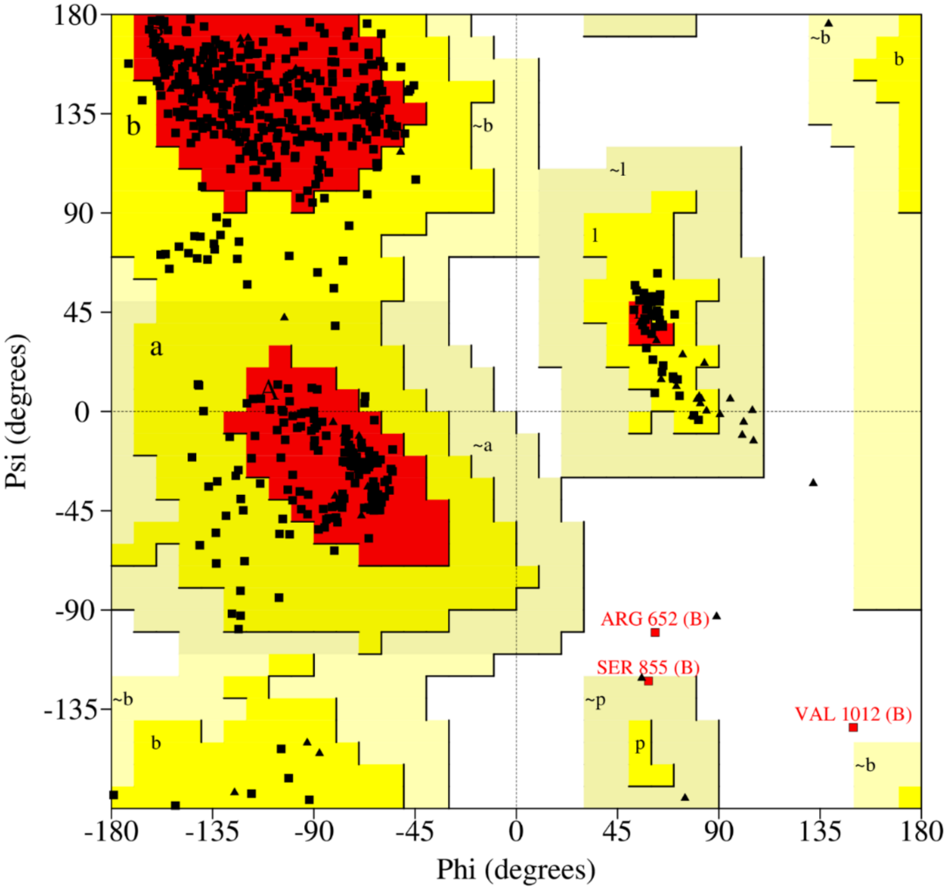

Supplementary Figure 10. Ramachandran plot for the LPR5 (PE3-PE4) homology model structure. 82.5% of the residues are present in the most favored region, and 16.9% of the residues are in the additionally allowed region. A total of 99.4% of the residues in the allowed regions indicate the good stereochemical quality of the structure.

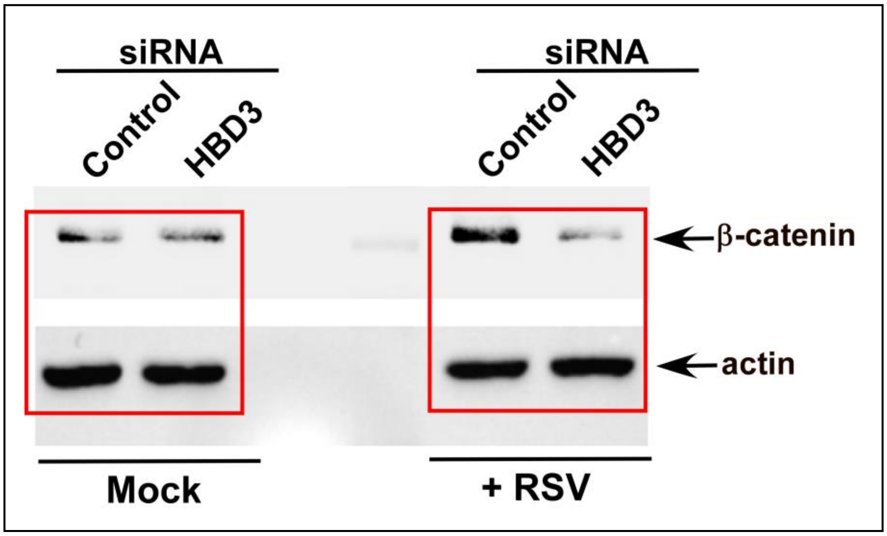

Supplementary Figure 11. The non-spliced blot related to Figure 6C is shown above. The portions of the blot shown in Figure 6C are indicated above with red boxes.
